# Evaluation of Metagenomic-Enabled Antibiotic Resistance Surveillance at a Conventional Wastewater Treatment Plant

**DOI:** 10.3389/fmicb.2021.657954

**Published:** 2021-05-13

**Authors:** Haniyyah J. Majeed, Maria V. Riquelme, Benjamin C. Davis, Suraj Gupta, Luisa Angeles, Diana S. Aga, Emily Garner, Amy Pruden, Peter J. Vikesland

**Affiliations:** ^1^Department of Civil & Environmental Engineering, Virginia Polytechnic Institute and State University, Blacksburg, VA, United States; ^2^Interdisciplinary Ph.D Program in Genetics, Bioinformatics, and Computational Biology, Virginia Polytechnic Institute and State University, Blacksburg, VA, United States; ^3^Department of Chemistry, University at Buffalo, Buffalo, NY, United States

**Keywords:** shotgun sequencing, resistome, microbiome, antibiotics, antibiograms, resistome (ARGs and MGEs)

## Abstract

Wastewater treatment plants (WWTPs) receive a confluence of sewage containing antimicrobials, antibiotic resistant bacteria, antibiotic resistance genes (ARGs), and pathogens and thus are a key point of interest for antibiotic resistance surveillance. WWTP monitoring has the potential to inform with respect to the antibiotic resistance status of the community served as well as the potential for ARGs to escape treatment. However, there is lack of agreement regarding suitable sampling frequencies and monitoring targets to facilitate comparison within and among individual WWTPs. The objective of this study was to comprehensively evaluate patterns in metagenomic-derived indicators of antibiotic resistance through various stages of treatment at a conventional WWTP for the purpose of informing local monitoring approaches that are also informative for global comparison. Relative abundance of total ARGs decreased by ∼50% from the influent to the effluent, with each sampling location defined by a unique resistome (i.e., total ARG) composition. However, 90% of the ARGs found in the effluent were also detected in the influent, while the effluent ARG-pathogen taxonomic linkage patterns identified in assembled metagenomes were more similar to patterns in regional clinical surveillance data than the patterns identified in the influent. Analysis of core and discriminatory resistomes and general ARG trends across the eight sampling events (i.e., tendency to be removed, increase, decrease, or be found in the effluent only), along with quantification of ARGs of clinical concern, aided in identifying candidate ARGs for surveillance. Relative resistome risk characterization further provided a comprehensive metric for predicting the relative mobility of ARGs and likelihood of being carried in pathogens and can help to prioritize where to focus future monitoring and mitigation. Most antibiotics that were subject to regional resistance testing were also found in the WWTP, with the total antibiotic load decreasing by ∼40–50%, but no strong correlations were found between antibiotics and corresponding ARGs. Overall, this study provides insight into how metagenomic data can be collected and analyzed for surveillance of antibiotic resistance at WWTPs, suggesting that effluent is a beneficial monitoring point with relevance both to the local clinical condition and for assessing efficacy of wastewater treatment in reducing risk of disseminating antibiotic resistance.

## Introduction

Antibiotic resistance is a complex health threat that requires both global and local action. Wastewater treatment plants (WWTPs) are a promising point of surveillance and mitigation, as they receive a confluence of sewage containing antibiotics, other antimicrobials, antibiotic resistant bacteria (ARB), antibiotic resistance genes (ARGs), and pathogens (Bürgmann et al., 2018). However, clear guidance is lacking with respect to which stage(s) of wastewater treatment and which monitoring targets are most informative with respect to assessing the ARG content of a given sewage. Of particular interest are the potential for ARGs to mobilize and spread and the efficacy of WWTPs in reducing ARG loads and associated human health risks prior to discharge or reuse (Aarestrup and Woolhouse, 2020).

Recent research demonstrates that ARGs that enter a given WWTP are reflective of various attributes of the local population, including antibiotic use patterns and socioeconomic factors (Hendriksen et al., 2019). These ARGs may exist on mobile genetic elements (MGEs), such as plasmids and transposons (Kim et al., 2014), which can facilitate their spread between different bacteria, including human pathogens. Further, ARGs may vary in clinical relevance of the antibiotic to which they encode resistance (e.g., front-line versus last-resort antibiotics) and may exist intracellularly (i.e., within ARB) or extracellularly as naked DNA that could potentially be later assimilated via transformation (Woegerbauer et al., 2020). Ideally, an effective surveillance scheme will serve to capture the breadth and depth of the full ARG profile as it changes through each stage of treatment, while also providing the ability to link the observed ARG patterns to clinical antibiotic resistance concerns, both on a local and global scale (Huijbers et al., 2019).

Conventional WWTPs rely on the dense, highly microbially active biological treatment step of activated sludge to efficiently remove organic matter and attenuate pathogens present in sewage. This reliance on activated sludge has brought about concern that this stage of treatment may serve as a “hotspot” for the mobilization of ARGs (Baquero et al., 2008; Zhang et al., 2009; Rizzo et al., 2013). This concern largely stems from the potential for the conditions within activated sludge to facilitate horizontal transfer of ARGs to pathogenic host bacteria (Zhang et al., 2011). Numerous studies have reported tracking ARB and ARGs through WWTPs (Bréchet et al., 2014; Yang et al., 2014; Mao et al., 2015; Guo et al., 2017; Joseph et al., 2019; Ju et al., 2019). Shotgun metagenomic sequencing is a promising means to gain such insight, as it enables direct profiling of total ARGs representative of a given sample (i.e., the “resistome,” Wright, 2007), without biases associated with culture or primer-directed gene amplification methods (e.g., quantitative polymerase chain reaction (qPCR) or qPCR array).

A challenge of metagenomic surveillance approaches for antibiotic resistance is that they support generation of data for up to thousands of ARGs, making it difficult to systematically and meaningfully assess and compare resistomes, both locally for a given WWTP with time, and globally with other WWTPs (Aarestrup and Woolhouse, 2020). The quantitative capacity of metagenomics is also not well defined, which is important for informing human health risk assessment (Manaia et al., 2018). Furthermore, consensus is lacking with respect to ideal locations within the WWTP to sample, frequency of sampling, and which ARG targets are most informative with respect to potential for ARGs to mobilize, efficacy of treatment for reducing ARGs, and relevance to human health risk assessment. A few recent studies have attempted to classify sewage (Hendriksen et al., 2019; Pärnänen et al., 2019) and final effluent (Pärnänen et al., 2019) by geographical region to predict antibiotic resistance burden. Comprehensive metagenomic analysis within individual WWTPs over time and comparison with trends relative to other WWTPs can help clarify which ARGs and groups of ARGs are most informative for linking to clinical resistance in a given community, for assessing WWTP ARG removal performance, and identifying potential anomalies that warrant further attention. Given the monetary cost of metagenomic sequencing, comprehensive longitudinal studies of WWTPs can help to narrow down key sampling locations, sampling frequencies, and replication needed for global comparative studies.

The overarching objective of this study was to comprehensively assess the composition of the resistome through the various stages of treatment and with time at a conventional WWTP to identify candidate metagenomic-derived targets for antibiotic resistance surveillance. This was achieved through eight sampling events over an 18-month period at a local WWTP followed by metagenomic sequencing. These samples were then compared and bench-marked to various dimensions of the resistome as they related to independent quantitative measures of target ARGs, factors associated with antibiotic use in the community and ARG selection (e.g., antibiotics), mobility (e.g., associations with MGEs), and local clinical resistance information. Specifically, we examined the core resistome (i.e., the full complement of ARGs detectable across all treatment stages), discriminatory resistomes (i.e., ARGs that separate the influent from effluent), specific ARGs of clinical concern, and resistome risk scores (i.e., the extent to which ARGs are predicted to occur on MGEs and in pathogens; Martínez et al., 2015; Oh et al., 2018) and compared these to independent qPCR measurements of target ARGs, antibiotic measurements, and local clinical resistance data. The findings provide insight into informative sampling locations, frequencies, and targets suitable for monitoring of antibiotic resistance flowing into and emanating from WWTPs.

## Materials and Methods

### Site Description, Sample Collection, and Sample Preservation

A three million gallons per day (MGD), on average, conventional (anoxic/oxic process with enhanced nitrogen removal) WWTP serving a population of approximately 21,500 in southwestern Virginia, United States was subjected to approximately bi-monthly sampling over the course of 18 months. The design capacity of the WWTP is six MGD, treating approximately 95% municipal wastewater (as % of COD) and 5% pre-treated industrial wastewater. Two industries contribute to the inflow at the WWTP: (1) a machine and fabrication plant discharging an average of 15,000 gallons per day (GPD) and (2) an environmental waste industry discharging an average of 1,600 GPD out of an allowed 14,000 GPD (varies greatly depending on weather conditions). Prior to discharge to a local river, the final effluent is subject to ultraviolet disinfection, with secondary effluent samples collected prior to UV and final effluent samples collected following this treatment step. To explore seasonality in subsequent analyses, samples were separated from the influent (*n* = 8) and secondary effluent (*n* = 8) and containing two sampling events in each season. Influent and secondary effluent samples were also separated into grouped seasons as such: winter/spring and summer/fall to capture a gradual transition from one extreme season (i.e., summer or winter) to the next. Details about the eight sampling events are provided in Supplementary Table 1.

Grab samples were collected using sterile materials at each stage of treatment and stored on ice until further processing. Temperature, dissolved oxygen (DO), and pH were measured on site for each aqueous sample. Upon receipt at the lab, aqueous samples were divided in triplicate by mixing vigorously and subsequently measuring the same mass of water for each replicate prior to concentrating on a 0.22-μm mixed-cellulose ester membrane filter (Millipore, Billerica, MA, United States). The volume of water (determined by mass) for the first replicate to clog the filter was recorded. Filters were folded and stored in a 50% ethanol solution in 2-mL O-ring tubes and preserved at −20°C. At a later date, the filters were torn into ∼0.1 cm^2^ pieces using sterile forceps, transferred to lysing tubes and DNA extraction was performed using a FastDNA SPIN Kit for Soil (MP Biomedicals, Solon, OH, United States).

### Shotgun Metagenomic Analysis

Twenty-two samples were selected for shotgun metagenomic sequencing, with pooling of triplicate DNA extracts in equal mass proportions. These included influent and secondary effluent (i.e., prior to disinfection) samples from each sampling event and a cross section of each WWTP process (i.e., influent, primary effluent, activated sludge, secondary effluent, and final effluent) for representative summer (August 2018) and winter (February 2018) events. Sequencing was performed by Diversigen, Inc. (Houston, TX, United States) on an Illumina NovaSeq 6000 utilizing the NexteraXT DNA Flex library preparation kit (Illumina, San Diego, CA, United States). The target depth was 7 gigabases per sample, corresponding to approximately 47 million reads (2 × 150 paired-end). Metagenomic read statistics are detailed in Supplementary Table 2. The samples were uploaded to the MetaStorm (Arango-Argoty et al., 2016) pipeline whereby they were quality filtered prior to annotation with the following databases: Comprehensive Antibiotic Resistance Database (CARD) version 2.0.1 (Jia et al., 2017) and Metagenomic Phylogenetic Analysis 2 (MetaPhlAn2) (Truong et al., 2015). The manual curation of CARD used in this study, as described in the Supplementary Material, can be found in the Supplementary Data 1. CARD output from MetaStorm is available in Supplementary Data 6, reported as relative abundance (i.e., ARG copies per copies of 16S rRNA genes identified from metagenomic data; Li et al., 2015). To determine calculated absolute abundance (i.e., ARG copies/mL; Supplementary Data 7), relative abundance values were multiplied by 16S rRNA gene copies as determined via qPCR (Garner et al., 2018). To help inform monitoring targets, four categories of ARGs were specified within the core resistome according to their absolute differences in the influent and effluent: **(Category 1)** detected only in the influent across all sampling events, **(Category 2)** increased in the secondary effluent with respect to the influent across all sampling events, **(Category 3)** decreased across all sampling events in the secondary effluent with respect to the influent, or **(Category 4)** detected only in secondary effluent across all sampling events.

Reads were assembled in MetaStorm using the IDBA-UD *de novo* assembler (Peng et al., 2012) according to default parameters to generate contigs for gene contextualization and clinically relevant pathogen-ARG screening. Contigs were filtered for sequences ≥1000 bps then protein-coding open reading frames (ORFs) were predicted using Prodigal version 2.6.3 with the “-p meta” option (Hyatt et al., 2010). Predicted ORFs were annotated with CARD and an in-house constructed MGE dataset (Arango-Argoty et al., 2019) using blastp in Diamond version 0.9.24 (Buchfink et al., 2015). Diamond alignments were filtered for stringent ARG and MGE annotation (80% identity, aa length ≥ 100, e-value ≤ 1e-10, bitscore ≥ 50). Each contig was assigned taxonomy using Kraken2 version 2.0.7 (Wood and Salzberg, 2014) with the Kraken2 standard database of complete bacterial, archaeal, and viral genomes in RefSeq.

The core resistome of the influent and secondary effluent was determined as any ARG with a non-zero value relative abundance detected across all sampling events. ExtrARG (Gupta et al., 2019), established based on the extremely randomized tree algorithm, was utilized to identify discriminatory ARGs (i.e., ARGs that collectively distinguish different wastewater samples) taking relative abundance into account.

### Assessment of Relative Resistome Risk

Contigs were submitted to the MetaCompare pipeline (Oh et al., 2018). MetaCompare assigns relative resistome risk (i.e., the cumulative potential for ARGs to occur on MGEs and in human pathogens, as inferred from assembled metagenomic data; Martínez et al., 2015) scores based on the distance each sample point is from an established theoretical, maximum risk score in a 3-dimensional “hazard space.” A sample’s location in this 3-dimensional space is determined as: (1) the number of occurrences of ARGs on assembled contigs, (2) the number of co-occurrences of ARGs and MGEs, and (3) the number of co-occurrences of ARGs, MGEs, and human pathogen-like sequences, all of which were normalized to the total number of contigs.

### Local, Clinical Resistance Isolates

According to the Virginia Healthcare Emergency Management Program, the WWTP in this study is located in the “Near Southwest” region. Fourteen out of fifteen hospitals from this region participated in the 2017 Virginia state and regional cumulative antibiogram (Virginia Department of Health, 2017). The 2017 regional data was used to identify the number of resistant isolates by antibiotic and resistance class corresponding to each species tested.

### Quantitative Polymerase Chain Reaction

All qPCR assays were performed on a CFX96 Real Time System (BioRad, Hercules, CA, United States) on triplicate DNA extracts. Gene copies of total bacterial 16S rRNA genes (Suzuki et al., 2000) and the following indicators ARGs of anthropogenic and clinical relevance were quantified in triplicate reactions based on previously published protocols: *bla*TEM (Bibbal et al., 2007), *erm*B (Chen et al., 2007), *sul*1 (Pei et al., 2006), *van*A (Dutka-Malen et al., 1995), and *int*I1 (Hardwick et al., 2008). A 100-fold dilution was determined as optimal to minimize inhibition and applied to the corresponding influent, secondary, and final effluent DNA extracts. On each qPCR plate, a triplicate negative control and standard curve ranging from 10^7^ to 10^1^ gene copies/μL were included for each target gene. Melt curves were assessed to confirm specificity of the amplicons and standards. Primers, *R*^2^ values, and efficiencies of the standard curves of each assay are reported in Supplementary Table 9.

### Antibiotic Analysis

Aqueous samples were concentrated using solid phase extraction, and the cartridges shipped to the University at Buffalo for analysis using high performance liquid chromatography-tandem mass spectrometry (HPLC-MS/MS), as described previously (Singh et al., 2019). The analysis included the following antibiotics: acetylsulfamethoxazole, anhydro erythromycin, anhydrochlorotetracycline, azithromycin, chlorotetracycline, ciprofloxacin, clarithromycin, erythromycin, enrofloxacin, norfloxacin, oxolinic acid, oxytetracycline, roxithromycin, sarafloxacin, spiramycin I, spiramycin II, spiramycin III, sulfachlorpyridazine, sulfadiazine, sulfamethoxine, sulfamerazine, sulfamethazine, sulfamethizole, sulfamethoxazole, sulfamethoxydiazine, sulfathiazole, tetracycline, tilmicosin, trimethoprim, and tylosin. Water samples were collected in pre-combusted (at a temperature of 500°C) amber glass bottles. Because sample collection and solid phase extraction protocols required the use of amber glass bottles, the ability to detect tetracyclines was lost due to their tendency to sorb to glass.

### Statistical Analysis and Data Visualization

To determine differences between stages of treatment, a paired Wilcoxon rank-sum test was utilized using the wilcox.test in R version 3.5.1 (R Core Team, 2018) with the built-in stats package. To compare differences among groups based on sampling event, stage of treatment, and seasonality one-way analysis of similarities (ANOSIM) based on Bray–Curtis dissimilarity was conducted in R using the anosim function of the *vegan* (Oksanen et al., 2019) R package. Procrustes analysis was conducted using the procrustes function in the *vegan* R package. Correlation analyses were performed using the Spearman option in rcorr contained within the *Hmisc* R package. A significance level of α = 0.05 was used for all statistical analyses. Coefficients of variation, utilized to assess relative variability on resistance class abundance in the influent or secondary effluent, was calculated by dividing the standard deviation by the mean. Nonmetric multi-dimensional scaling analysis was applied to visualize and compare relative abundances of ARGs and taxonomic ranks across samples. Graphics were generated using the *ggplot2* (Wickham, 2016), *circlize* (Gu et al., 2014), and *RColorBrewer* (Neuwirth, 2014) packages in R.

## Results

### Wastewater Physiochemical Parameters

Correlations were examined between several wastewater physiochemical parameters measured at the time of sampling or monitored by the WWTP (e.g., daily TSS measurements, Supplementary Table 1). Influent water temperature, ambient temperature, DO, pH, and TSS were not strongly correlated with any individual ARGs (Supplementary Data 5).

### Assessment of Metagenomic Sequencing Data and Comparison to qPCR

Metagenomic sequencing yielded an average of approximately 37 million (range: 3.7–54.5 million) paired-end reads per sample (Supplementary Table 2). A total of 953 ARGs were identified across all 22 samples collected over the eight sampling events. A Spearman rank order correlation analysis was performed to explore the relationship between ARG abundance as determined by qPCR (Supplementary Figure 1) and the calculated absolute abundance (i.e., ARG copies/mL) of ARGs derived from metagenomic data (Supplementary Data 7). Significant correlations were observed for *erm*B (*R* = 0.87, Bonferroni-corrected *p* < 0.001), *sul*1 (*R* = 0.83, Bonferroni-corrected *p* < 0.001), and aggregate *bla*TEM genes (*bla*TEM-17, *bla*TEM-57, *bla*TEM-75, *bla*TEM-91, *bla*TEM-166, *bla*TEM-176, *bla*TEM-194, *bla*TEM-195, *bla*TEM-207, *bla*TEM-215) (*R* = 0.84, Bonferroni-corrected *p* < 0.001) versus corresponding *erm*B, *sul*1, and *bla*TEM measurements by qPCR. *van*A, a vancomycin ARG, was only detected by metagenomics in one effluent sampling event (December 2017) and therefore could not be correlated to qPCR data. These results supported further quantitative evaluation of metagenomically derived measurements of the resistome.

### Trends in ARG Abundance Detected in Influent and Effluent

**FIGURE 1:**
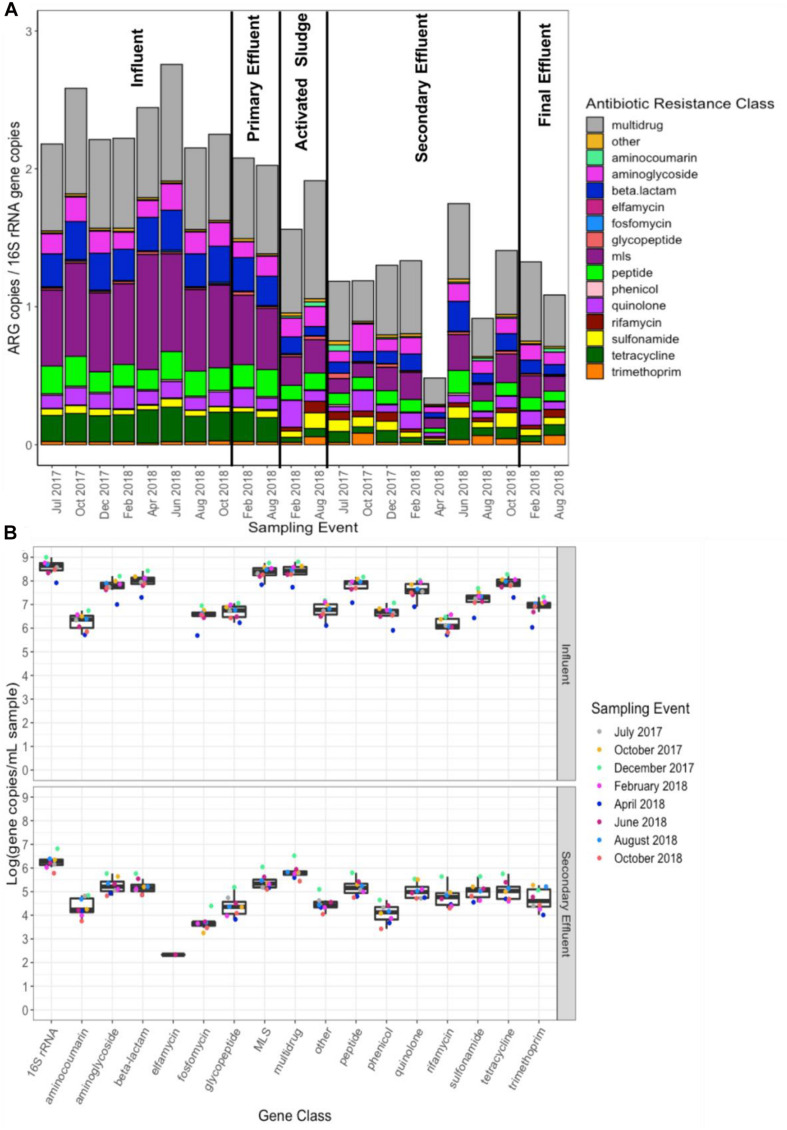
**(A)** Total ARG (i.e., all ARGs detected) relative abundance [copies per copies of 16S rRNA normalized as previously described (Li et al., 2015)], grouped by antibiotic resistance class, across all 22 samples subject to shotgun metagenomic sequencing and **(B)** calculated absolute abundance (units: log_10_[gene copies/mL sample]; relative abundance multiplied by 16S rRNA gene copies quantified by qPCR) of each resistance class. ARGs were identified via annotation against CARD version 2.0.1 (Jia et al., 2017). “Multi-drug” represents ARGs conferring resistance to antibiotics corresponding to at least two drug classes, whereas the “other” category comprises genes conferring resistance to non-antibiotics (e.g., antimicrobials, antifungals). MLS indicates resistance to macrolides, lincosamides, and streptogramins.

There were 859 ARGs detected across all influent samples and an average total ARG (i.e., all ARGs detected) relative abundance (i.e., normalized per 16S rRNA gene copies) of 2.35 (minimum = 2.15, maximum = 2.76). ARGs corresponding to the following classes represented the majority of ARGs detected: multidrug (range: 26.7–30.7% of total ARGs), macrolide-lincosamide-streptogramin (MLS, 25.3–34.1%), and beta-lactam (9.9–12.4%) (Figure 1). Across all secondary effluent samples, there were 637 ARGs detected and an average total ARG relative abundance of 1.20 (minimum = 0.48, maximum = 1.75). ARGs corresponding to the following resistance classes were most abundant: multidrug (range: 24.7–39.6% of total ARGs), MLS (8.6–14.9%), aminoglycoside (6.6–16.7%), beta-lactam (6.1–12.4%) and quinolone (3.0–12.4%) (Figure 1).

ANOSIM confirmed that there was a distinct shift in the resistome composition from influent to effluent, while there was no significant separation between the secondary versus final effluent (Supplementary Tables 4, 5). Focus on secondary effluent provides a more consistent DNA yield and presents the advantage of comparability across WWTPs, as not all WWTPs employ effluent disinfection. For each sampling event, there was a decrease in total ARG relative abundance from influent to secondary effluent (Wilcoxon, paired; *p* = 0.007813), corresponding to an average removal of 1.15 ARG copies/16S rRNA gene copies or approximately 50% reduction (Figure 1).

**FIGURE 2:**
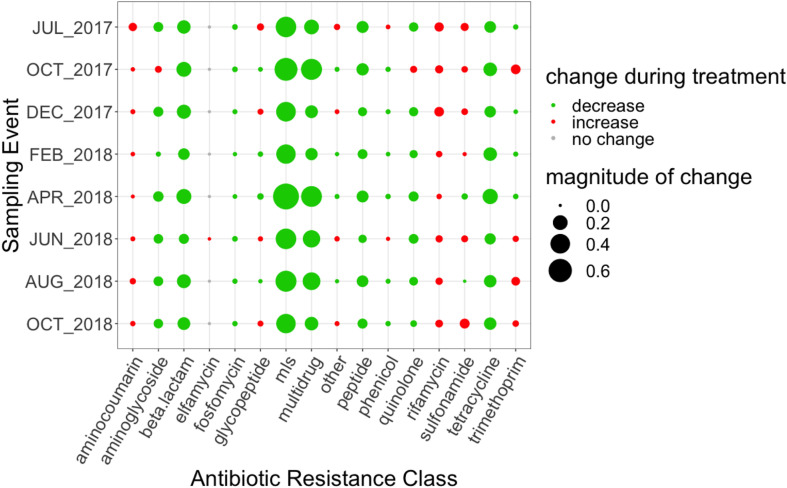
Magnitude of change (based on absolute difference) between influent and secondary effluent for each ARG grouped by antibiotic resistance class. Change was calculated as the difference between the relative abundance of the secondary effluent and the relative abundance of the influent. MLS indicates resistance to macrolides, lincosamides, and streptogramins.

The change in relative abundance between the influent to the secondary effluent by antibiotic resistance class ranged between 8.15 × 10^–5^ to 0.76 ARG copies/16S rRNA gene copies (Figure 2). The total number of mapped reads by resistance class in the influent and secondary effluent can be found in Supplementary Table 10. On average, the magnitude of observed changes was ∼2,200 reads. MLS ARGs decreased to the greatest extent, with an average removal of 0.49 ARG copies/16S rRNA gene copies. However, a net increase in the relative abundance of ARGs conferring resistance to several classes of antibiotics was indicated through the secondary clarification process for some sampling events (# events): aminocoumarin (8), glycopeptide (4), phenicol (2), rifamycin (8), sulfonamide (6), trimethoprim (4), and other (4) (Figure 2).

There was a similar magnitude of decrease in absolute abundance (i.e., ARG copies/mL) of various ARG classes across the WWTP (Figure 1). Absolute abundances were calculated from relative abundances of ARGs (ARGs/16S rRNA genes) based on independent measurements of 16S rRNA gene copies per mL via qPCR, which decreased more than 2-log_10_ from influent to secondary effluent (Figure 1).

### Core Resistome Composition and Behavior

**FIGURE 3:**
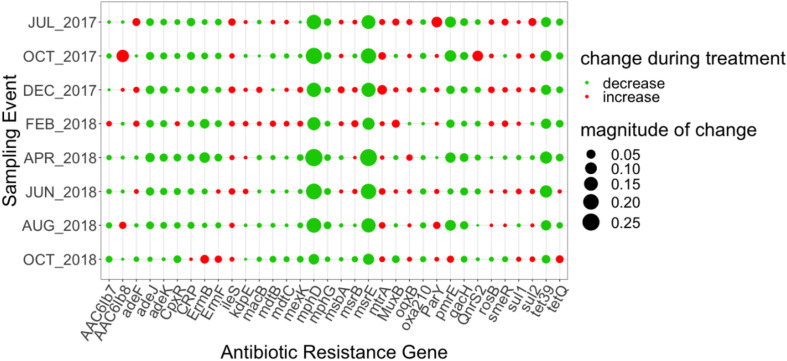
Magnitude of change (based on absolute difference) in relative abundance between influent and secondary effluent of top 34 ARGs pertaining to the core resistome (ARGs detected across each treatment process and all sampling events). Change was calculated as the difference between the relative abundance of the secondary effluent and the relative abundance of the influent.

The “core WWTP” resistome, defined as ARGs detected across each treatment process and all sampling events, consisted of 111 ARGs. However, when considering only the influent and secondary effluent, there were 143 ARGs identified across all sampling events. The 25 ARGs with the highest mean relative abundance in the influent and secondary effluent are presented in Tables 1, 2, respectively. There were 34 ARGs that overlapped between the top 25 ARGs in the influent and the top 25 ARGs in the effluent (Supplementary Figure 2). There was an increase in relative abundance of 26 of these 34 ARGs from influent to effluent in all or certain sampling events. For example, *sul*1 increased in six sampling events, excluding April 2018 and August 2018, when the relative abundance decreased. Likewise, the relative abundance of *sul*2 increased in six sampling events, excluding February 2018 and April 2018, when the relative abundance decreased. Interestingly, the calculated absolute abundances (i.e., ARG copies/mL) of *sul*1 and *sul*2 were perfectly correlated (*R* = 1.00, *p* < 0.001). February 2018 represented the sampling event with the greatest number of ARGs exhibiting an increase in relative abundance from influent to secondary effluent (Figure 3).

**TABLE 1:** Top 25 most abundant core influent ARGs (detected in all sampling events), based on mean relative abundance, that were also detected in secondary effluent core resistome determined by metagenomics.

Antibiotic resistance class	Antibiotic resistance genes
Aminocoumarin	*par*Y in *Streptomyces rishiriensis*
Aminoglycoside	*aac*(6′)-Ib7
Beta-lactam	*bla*OXA-210
MLS	*mac*B, *mph*D, *mph*G, *msr*B, *msr*E, *erm*B, *erm*F
Peptide	*pmr*E, *ros*B
Quinolone	*qac*H, *qnr*S2
Sulfonamide	*sul*1, *sul*2
Tetracycline	*tet*39, *tet*Q
Multidrug	*ade*J, *ade*K, *cpx*R in *Pseudomonas aeruginosa*, *crp*, *mdt*B, *mex*K, *msb*A, *mux*B

**TABLE 2:** Top 25 most abundant core secondary effluent ARGs (detected in all sampling events), based on mean relative abundance, that were also detected in the influent core resistome determined by metagenomics.

Antibiotic resistance class	Antibiotic resistance genes
Aminocoumarin	*par*Y in *Streptomyces rishiriensis*
Aminoglycoside	*aac*(6′)-Ib7, *aac*(6′)-Ib8, *kdp*E
MLS	*mac*B, *mph*D, *msr*B, *msr*E
Peptide	*pmr*E, *ros*B
Quinolone	*qac*H, *qnr*S2
Sulfonamide	*sul*1, *sul*2
Multidrug	*ade*F, *cpx*R in *Pseudomonas aeruginosa*, *crp*, *mdt*B, *mdt*C, mexK, *msb*A, *mux*B, *mtr*A, *sme*R, *oqx*B
Other	*ile*S in bifidobacteria

### Categorizing Core ARGs Based on Tendency to Increase or Decrease During Treatment

An average of approximately half (41.1–68.0%) of the total ARGs that entered the WWTP on any given sampling date persisted through the secondary effluent (Table 3). There were only a relatively small number of ARG types that appeared in the WWTP secondary effluent that were not detected in the influent. Notably (in 7/8 events), ∼90% of the ARG types detected in the secondary effluent were also found in the influent (i.e., were not removed by the treatment train). There were only two ARGs in Category 1: *aph*(3′)-VI (aminoglycoside) and *opr*Z (multidrug). Eleven ARGs fell into Category 2 belonging to the following classes: one beta-lactam (*bla*OXA*-*46), three rifamycin (*arr*-1, *rph*A, *rph*B), two tetracycline [*tap*, *tet*A(48)], four multidrug (*efp*A, *mex*N, *mtr*A, *mux*C), and one mupirocin, classified as “other” (*ile*S in bifidobacteria). Sixty-one ARGs were included in Category 3 (Supplementary Table 3) and no ARGs were included in Category 4 when collectively considering all sampling events. However, between 22 and 93 ARGs uniquely appeared in the secondary effluent when considering individual sampling dates. The greatest number of unique ARGs (i.e., highest diversity) that were detected in the secondary effluent, but not the influent, corresponded to the October 2018 sampling event. Thus, we analyzed the shared ARGs between the October 2018 sampling event and all other sampling events (Table 4). A substantial number of these shared ARGs encoded resistance to beta-lactams, although the total relative abundance of this resistance class decreased with each sampling event.

**TABLE 3:** ARGs detected in the influent only, effluent only, or shared between influent and effluent determined by metagenomics.

Sampling event (total ARGs in influent/effluent)	# of unique ARGs detected in the influent (% total ARGs in influent)	# of unique ARGs detected in the effluent (% total ARGs in effluent)	# of ARGs detected in influent *and* effluent (% total ARGs in influent/effluent)
July 2017 (576/271)	334 (58.0)	29 (10.7)	242 (42.0/89.3)
October 2017 (670/331)	318 (47.5)	39 (11.8)	292 (52.5/88.2)
December 2017 (596/330)	309 (51.8)	43 (13.0)	287 (49.2/87.0)
February 2018 (585/309)	298 (50.9)	22 (7.1)	287 (41.1/92.9)
April 2018 (546/326)	253 (46.3)	33 (10.1)	293 (53.7/89.9)
June 2018 (643/441)	250 (38.9)	48 (10.9)	393 (61.1/89.1)
August 2018 (551/381)	219 (39.7)	49 (12.9)	332 (60.3/87.1)
October 2018 (341/325)	109 (32.0)	93 (28.6)	232 (68.0/71.4)

**TABLE 4:** ARGs detected only in the effluent that were also detected in the October 2018 sampling, which yielded the highest number of unique ARG detections determined by metagenomics.

Sampling event (# ARGs shared with October 2018 sampling)	ARGs shared
July 2017 (8)	*bla*F, *bla*CARB-12, *bla*VIM-23, *bla*LRA-19, *bla*OXA-224, *bla*OXA-29, *dfr*B2, *vat*A
October 2017 (15)	*AAC*(6′)-IIb, *bla*F, *bla*IMP-19, *bla*IMP-44, *bla*LRA-10, *bla*VIM-23, *dfr*A2d, *dfr*B2, *dfr*B6, *ole*C, *srm*B, *sul*3, *van*M, *van*XO, *vat*A
December 2017 (16)	*AAC*(3)-IIIc, *abe*S, *arr*-3, *bla*F, *bla*IMP-19, *bla*LRA-10, *bla*LRA-19, *bla*VIM-23, *dfr*A15, *dfr*B2, *dfr*B6, *mfp*A, *ole*C, *tlr*C, *vat*A, *mur*A in *Chlamydia trachomatis*
February 2018 (8)	*bla*F, *bla*IMP-19, *bla*LRA-19, *bla*VIM-2, *bla*VIM-23, *fos*A7, *tet*(Y), *van*M
April 2018 (17)	*AAC*(6′)-IIb, *bla*F, *bla*FEZ-1, *bla*LRA-10, *bla*LRA-19, *bla*OXA-29, *bla*VIM-2, *bla*VIM-23, *dfr*A16, *dfr*B2, *dfr*B6, *iri, ole*B, *ole*C, *qnr*B72, *vat*I, *mur*A in *Chlamydia trachomatis*
June 2018 (11)	*AAC*(3)-IIIc, *bla*FEZ-1, *bla*IMP-19, *bla*LRA-10, *bla*OXA-29, *bla*VIM-2, *bla*VIM-23, *dfr*A2d, *dfr*B6, *ole*B, *sul*3
August 2018 (10)	*arr*-2, *arr*-3, *arr*-5, *bla*LRA-10, *dfr*B6, *ole*C, *qep*A, *qep*A3, *sul*3, *tet*B(48)

### Discriminatory Resistome Composition and Behavior

**FIGURE 4:**
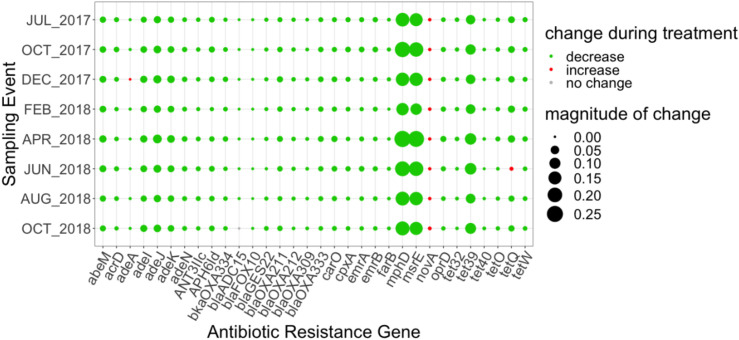
Magnitude of change (based on absolute difference) in relative abundance between influent and secondary effluent of ARGs (*n* = 32) detected in the discriminatory resistome (ARGs which most effectively distinguish the influent versus the secondary effluent) as determined by ExtrARG (Gupta et al., 2019). Change was calculated as the difference between the relative abundance of the secondary effluent and the relative abundance of the influent.

Thirty-two ARGs were found to most effectively distinguish the influent versus the secondary effluent based on their magnitude of change in relative abundance: one aminocoumarin (*novA*), three aminoglycoside (*acr*D, *ant*(3″)-IIc, *aph*(6)-Id), ten beta-lactam (*bla*ADC-15, *car*O, *bla*FOX*-*10, *bla*GES-22, *opr*D in *Acinetobacter baumannii, bla*OXA-211, *bla*OXA-212, *bla*OXA-309, *bla*OXA-333, *bla*OXA-334), two MLS (*mph*D, *msr*E), seven tetracycline (*ade*A, *tet*32, *tet*39, *tet*40, *tet*O, *tet*Q, *tet*W), eight multidrug (*abe*M, *ade*I, *ade*J, *ade*K, *ade*N, *emr*A, *emr*B), and *far*B, conferring resistance to antibacterial free fatty acids (other) (Figure 4). All of the aforementioned ARGs decreased in relative abundance from influent to effluent in each sampling event, except *nov*A and *tet*Q. *mph*D and *msr*E, which underwent the greatest decrease in relative abundance between influent and effluent, followed by *tet*39.

### Clinically Relevant ARGs

A database of 931 ARGs known in clinical isolates was compiled to help prioritize selection of core and discriminatory ARGs for further monitoring (Supplementary Data 2). Eleven clinically relevant ARGs (*bla*OXA-3, *bla*OXA-5, *bla*OXA-16, *bla*OXA-46, *bla*OXA-74, *bla*OXA-118, *bla*OXA-129, *bla*OXA-145, *bla*OXA-205, *bla*OXA-210, *qnr*S2) (Supplementary Table 7) were detected in the core resistome across all samples. Within the discriminatory resistome of the influent and secondary effluent, there were four clinically relevant ARGs (*bla*GES-22, *bla*OXA-212, *bla*OXA-309, *bla*OXA-333) (Supplementary Table 7) detected. Notably, 189 of the 954 total ARGs detected across all samples were also found in the database of clinically relevant ARGs.

### Shift in Microbiome Through the WWTP

**FIGURE 5:**
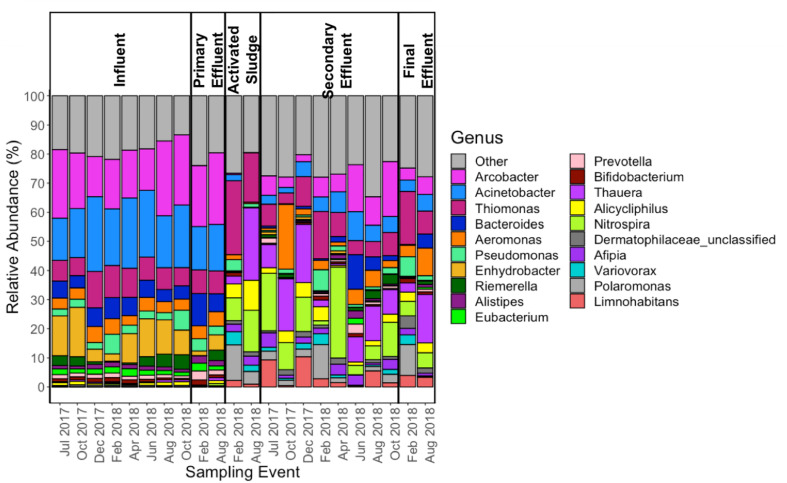
Relative abundance (as percentage) of the top 20 and “other” genera, as annotated using MetaPhlAn2 (Truong et al., 2015) across all 22 samples subject to metagenomic sequencing.

In most influent samples, *Arcobacter*, *Acinetobacter*, and *Enhydrobacter* comprised greater than 50% of the genera detected (Figure 5). This was not the case during the months of December 2017 and February 2018, when *Enhydrobacter* was much lower in relative abundance compared to other sampling events. While the influent and primary effluent were quite similar, there was a sharp shift in the composition of the microbial community in the activated sludge stage. Thereafter, the composition of the secondary effluent and final effluent mirrored that of the activated sludge (for the two events where activated sludge was analyzed. Some of the more dominant genera found from activated sludge onward included: *Thiomonas, Thauera, Nitrospira, Polaromonas*, and *Limnohabitans*. Notably, there appeared to be more variability in the abundance of the top 20 genera in activated sludge, secondary effluent, and final effluent as compared to influent and primary effluent (Figure 5).

### Trends in Taxonomic Annotations Corresponding to Locally Monitored Clinical Pathogenic Bacteria

**FIGURE 6:**
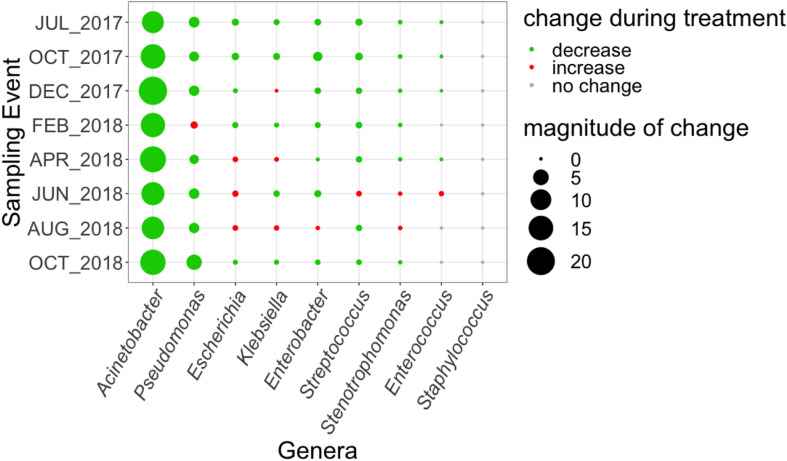
Magnitude of change (based on absolute difference) in relative abundance, as a percentage of corresponding genetic markers quantified from metagenomics data using MetaPhlAn2 (Truong et al., 2015) in influent versus secondary effluent, of pathogen-containing genera tested in the 2017 Virginia state and regional cumulative antibiogram (Virginia Department of Health, 2017).

As a means of linking the WWTP and local clinical data, we compared the abundance of genetic markers corresponding to the nine genera containing pathogens monitored by the regional antibiogram using MetaPhlAn2 (Truong et al., 2015) in the influent and secondary effluent across the eight sampling events (Figure 6). Genetic material corresponding to several of these organisms could still be found in the effluent, including: *Acinetobacter*, *Escherichia, Klebsiella, Enterobacter, Pseudomonas, Enterococcus, Streptococcus*, and *Stenotrophomonas*. Of these, all but *Acinetobacter* increased in relative abundance from influent to effluent in at least one sampling event.

### Variation of Resistome and Microbiome Through the WWTP and With Time

According to the coefficients of variation, as percent of the total ARG relative abundance of influent (9.1%) versus secondary effluent (30%) samples, the influent exhibited less variability in resistome composition with time than the secondary effluent (Table 5). Influent samples were consistently less variable than secondary effluent samples across all antibiotic resistance classes. Variability ranged from: 8.7–41% in the influent and from 30 to 91% in secondary effluent (Table 5).

**TABLE 5:** Coefficient of variation, as percent (%), in influent and secondary effluent metagenomic samples.

Antibiotic resistance class	Influent (*n*^a^ = 8)	Secondary effluent (*n*^a^ = 8)
Total ARG relative abundance	9.1	30
Aminocoumarin	24	91
Aminoglycoside	16	43
Beta-lactam	8.7	54
Elfamycin	ND^b^	NA^c^
Fosfomycin	22	66
Glycopeptide	25	67
MLS	15	42
Peptide	15	40
Phenicol	12	63
Quinolone	19	60
Rifamycin	41	48
Sulfonamide	21	45
Tetracycline	12	58
Trimethoprim	20	77
Multidrug	11	30
Other	20	34

*^a^*n* = number of samples. ^b^ARGs conferring resistance to this antibiotic class not detected in any samples. ^c^ARGs conferring resistance to this antibiotic class detected in only one sample.*

**FIGURE 7:**
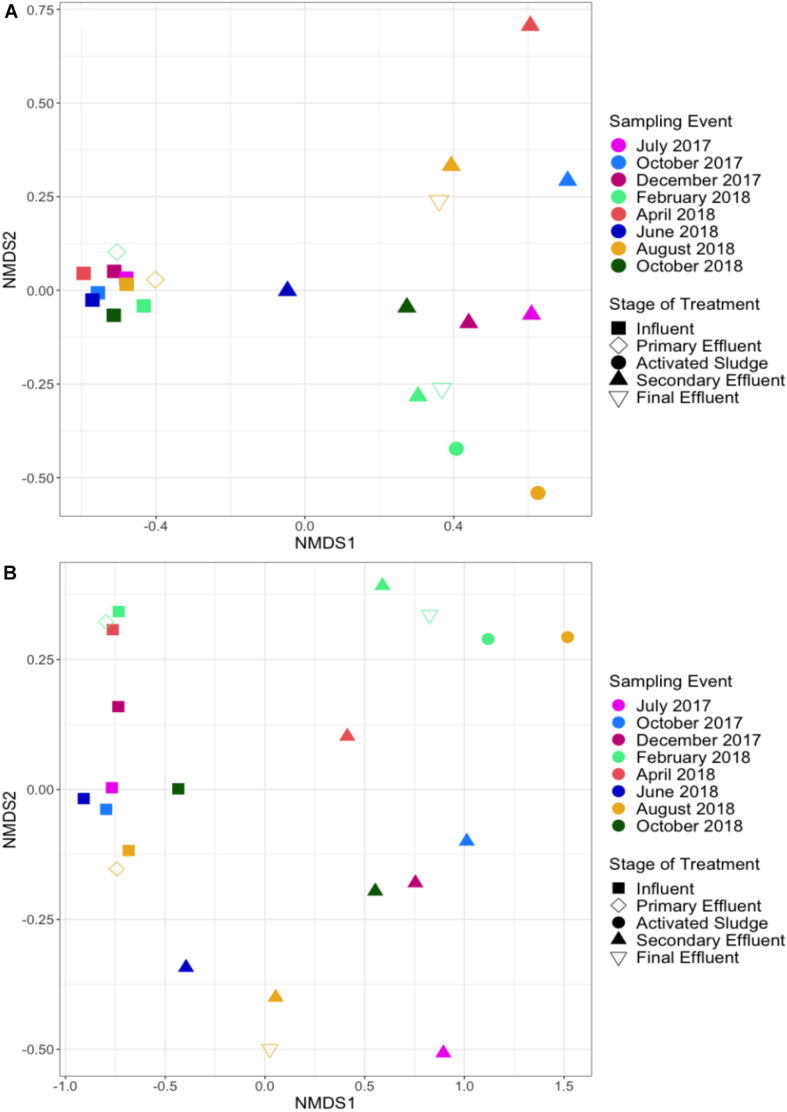
NMDS analysis of **(A)** ARG profiles (ANOSIM; *R* = 0.708, *p* = 0.001) and **(B)** taxonomic profiles (ANOSIM; *R* = 0.6547, *p* = 0.001) across WWTP sampling locations and sampling dates according to shotgun metagenomic sequencing. Influent samples were separated from activated sludge (ANOSIM; *R* = 1, *p* = 0.022), secondary effluent (ANOSIM; *R* = 0.933, *p* = 0.001), and final effluent (ANOSIM; *R* = 1, *p* = 0.022), but not from primary effluent (ANOSIM; *R* = 0.228, *p* = 0.178). Secondary and final effluent samples were not separated (ANOSIM; *R* = –0.306, *p* = 0.911). ARGs were annotated via CARD (Jia et al., 2017) and the microbiome was annotated via MetaPhlAn2 (Truong et al., 2015).

NMDS analysis of total ARG relative abundance indicated distinct resistomes associated with each stage of treatment (ANOSIM; #ARGs = 953, *R* = 0.708, *p* = 0.001; Figure 7). Influent samples were separated from activated sludge (ANOSIM; #ARGs = 953, *R* = 1, *p* = 0.022), secondary effluent (ANOSIM; #ARGs = 953, *R* = 0.933, *p* = 0.001), and final effluent (ANOSIM; #ARGs = 953, *R* = 1, *p* = 0.022), but not from primary effluent (ANOSIM; #ARGs = 953, *R* = 0.228, *p* = 0.178). Activated sludge samples were not separated from secondary effluent (ANOSIM; #ARGs = 953, *R* = 0.151, *p* = 0.267) or final effluent (ANOSIM; #ARGs = 953, *R* = 0.25, *p* = 0.333). The ANOSIM R statistic and significance level of each pairwise test between each stage of treatment based on relative abundance is available in Supplementary Table 4. ARGs that were not detected in at least one sample in a subset were excluded from ANOSIM analysis. When only influent and secondary effluent samples were grouped by stage of treatment, ARG profiles were separated based on relative abundance (ANOSIM; #ARGS = 916, *n* = 8, *R* = 0.933, *p* = 0.001).

Taxonomic profiles at the genus level followed a similar trend as ARGs and were also uniquely separated by stage of treatment (ANOSIM; *R* = 0.6547, *p* = 0.001). Influent samples were separated from activated sludge (ANOSIM; *R* = 1, *p* = 0.019), secondary effluent (ANOSIM; *R* = 0.801, *p* = 0.002), and final effluent (ANOSIM; *R* = 1, *p* = 0.024), but not from primary effluent (ANOSIM; *R* = 0.31, *p* = 0.136). Activated sludge samples were not separated from secondary effluent (ANOSIM; *R* = 0.289, *p* = 0.15) or final effluent (ANOSIM; *R* = 0.25, *p* = 0.667). The ANOSIM R statistic and significance level of each pairwise test between each stage of treatment based on genus level relative abundance is available in Supplementary Table 5.

A Procrustes analysis was performed on the ARG and taxonomic NMDSs of all 22 samples, assuming symmetry, resulting in a Procrustes Sum of Squares value equal to 0.261 (*R* = 0.86, *p* = 0.001). This low value suggests that ARG and taxonomic profiles behave similarly across the WWTP with time. Influent samples did not exhibit distinct separation based on relative abundance of ARGs when grouped by season (ANOSIM; #ARGS = 859, *R* = 0.1458, *p* = 0.241) or grouped seasons (ANOSIM; #ARGS = 859, *R* = 0.2396, *p* = 0.064). Abundance of genera also did not exhibit distinct separation based on grouped seasons (ANOSIM; *R* = 0.1354, *p* = 0.213). However, more distinct separation of the taxonomic profile as a whole was observed by season (ANOSIM; *R* = 0.5208, *p* = 0.04). Similar trends of no distinct separation of resistome or microbiome were observed based on relative abundances in secondary effluent samples: ARG profiles grouped by season (ANOSIM; #ARGS = 637, *R* = −0.1667, *p* = 0.785), ARG profiles based on grouped seasons (ANOSIM; #ARGS = 637, *R* = −0.04167, *p* = 0.63), taxonomic profiles grouped by season (ANOSIM; *R* = −0.1458, *p* = 0.775), and taxonomic profiles based on grouped seasons (ANOSIM; *R* = −0.1146, *p* = 0.789).

### Relative Resistome Risk

**FIGURE 8:**
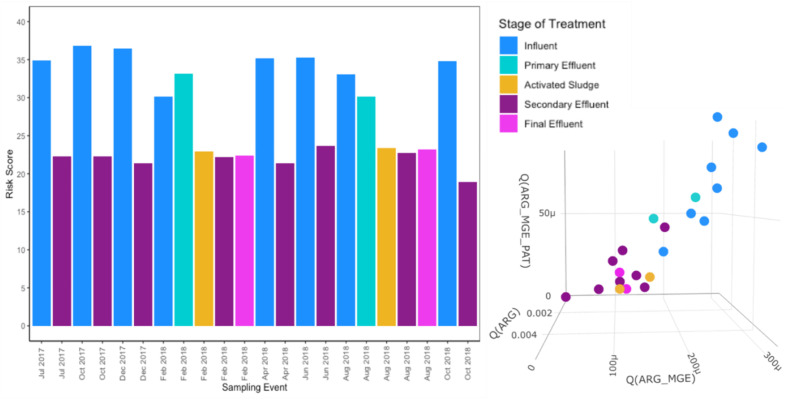
Resistome risk scores and projection in 3D hazard space for each sample as determined by MetaCompare (Oh et al., 2018) using assembled metagenomic data. Risk scores are calculated using: (1) the number of occurrences of ARGs on assembled contigs normalized to the total number of contigs in a given sample, (2) the number of co-occurrences of ARGs and MGEs normalized to the total number of contigs, and (3) the number of co-occurrences of ARGs, MGEs, and human pathogen-like sequences normalized to the total number of contigs.

As would be expected based on the WWTP achieving its intended purpose of reducing pathogens, MetaCompare indicated that there was a higher resistome risk associated with influent and primary effluent samples relative to activated sludge, secondary effluent, and final effluent (Figure 8). Influent relative resistome risk scores were significantly higher than those of the secondary effluent (Wilcoxon, paired; *p* = 0.007813). Overall, there was a consistent and high rate of metagenomic assembly incorporated into the resistome risk score determination (Supplementary Table 6). The percent of sequences successfully assembled across samples ranged from 24 to 69% (only four samples with <40%). A higher percent assembly was generally achieved for secondary effluent samples (Kruskal–Wallis, *p* = 0.0229).

### Comparison to Locally Available Clinical Resistance Data

Overall, there were 45 co-occurrences of ARGs, MGEs, and human pathogen-like sequences on assembled contigs recovered from the influent and 38 from the effluent that corresponded to antibiotic resistant pathogens tested in the regional antibiogram (Supplementary Data 3). Among the assembled contigs, there were none found in both the influent and effluent. Nonetheless, the same resistance classes associated with human pathogen-like sequences were found in the effluent, including tetracycline resistance in *E. faecium* and *S. aureus*, beta-lactam resistance in *K. pneumoniae*, and aminoglycoside resistance in *P. aeruginosa*. Multidrug resistance genes were excluded from subsequent analysis because antibiogram data were only available based on single organism-antibiotic combinations.

**FIGURE 9:**
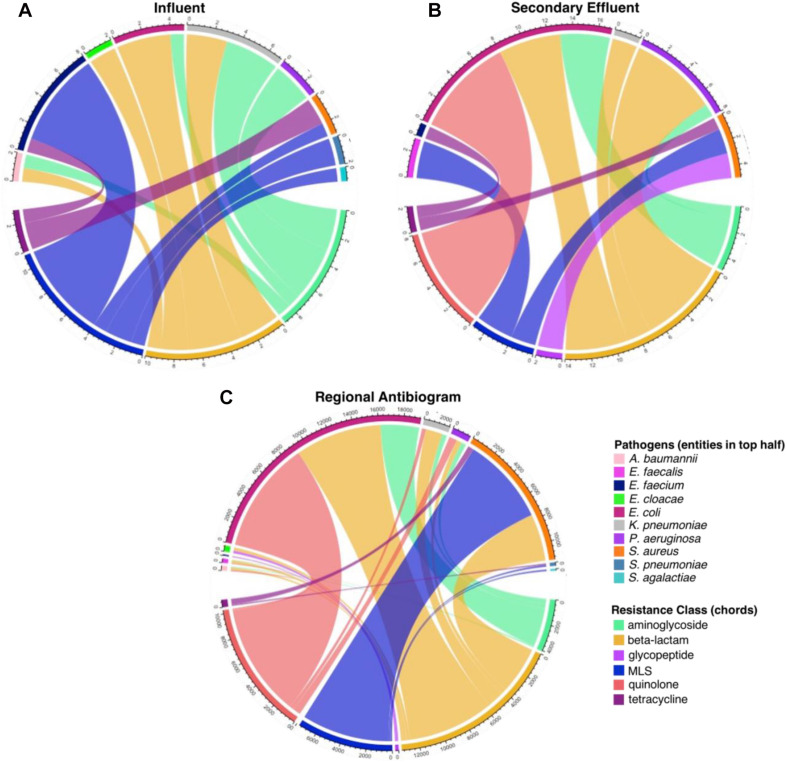
Comparison of ARG-pathogen combinations identified in the assembled metagenomic data and in the 2017 corresponding state and regional cumulative antibiogram (Virginia Department of Health, 2017). The values on the outer rings indicate the frequency of co-occurring antibiotic resistance genes (ARGs) grouped by resistance class, mobile genetic elements (MGEs), and human pathogen-like sequences in all **(A)** influent and **(B)** secondary effluent samples (Supplementary Data 3). **(C)** The values on the outer ring represent each antibiotic-pathogen combination which was tested in the regional antibiogram, aggregated by antibiotic resistance class (i.e., a single connection is indicative of one isolate conferring resistance to one antibiotic within a given class; Supplementary Data 4).

The greatest number of co-occurrences of ARGs with taxonomic markers of antibiogram pathogens in the influent were approximately equal among the MLS (11 co-occurrences), beta-lactam (10 co-occurrences), and aminoglycoside (9 co-occurrences) resistance classes (Figure 9). *E. faecium* contained the greatest number of co-occurrences, associated with glycopeptide and MLS resistance classes, followed by *K. pneumoniae*, associated with aminoglycoside and beta-lactam resistance classes. In contrast, the effluent was largely dominated by beta-lactam (14 co-occurrences) and quinolone (8 co-occurrences) resistance classes, with fewer co-occurrences associated with aminoglycoside and MLS resistance classes. *E. coli* comprised the greatest number of co-occurrences in the effluent and, unlike in the influent, was associated with quinolone, beta-lactam, and aminoglycoside resistance classes (in descending order). Remarkably, the trends apparent in the effluent were more similar to those of the regional antibiogram data than those apparent in the influent, especially the aforementioned associations with *E. coli* and MLS resistance in *S. aureus* (Figure 9). The number of resistant isolates determined from the regional antibiogram can be found in Supplementary Data 4.

### Antibiotic Detection and Correlation With Corresponding ARG Classes

**FIGURE 10:**
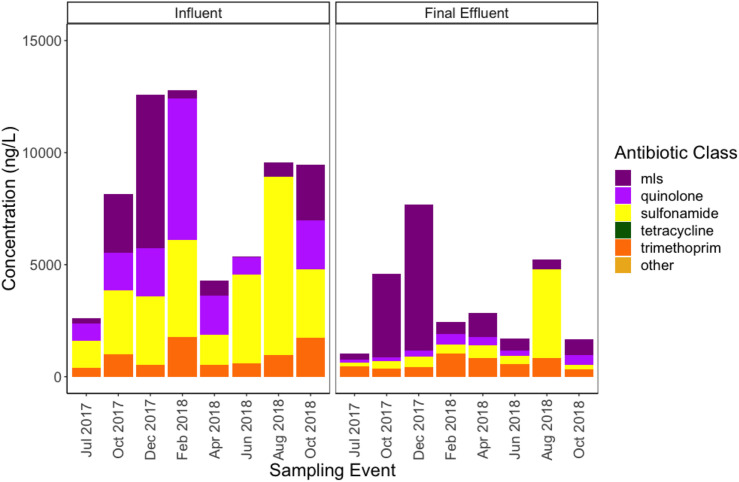
Concentrations of antibiotics (ng/L) detected via high performance liquid chromatography-tandem mass spectrometry in influent and final effluent samples grouped by resistance class. The methods applied were as described previously (Singh et al., 2019). MLS indicates antibiotics belonging to the macrolide, lincosamide, and streptogramin classes. Tetracycline and other (i.e., tylosin) antibiotics were tested but not detected in wastewater samples.

All antibiotic classes tested in the regional antibiogram were detectable in the WWTP samples, except beta-lactams, due to analytical challenges. Analysis of targeted antibiotics indicated a tendency to reduce from the influent to the final effluent (Figure 10 and Supplementary Table 8). However, among the antibiotics tested, the MLS and trimethoprim antibiotics appeared to be the most persistent and difficult to remove. Cumulative total measured antibiotic loading in the influent ranged between 2,614 and 12,780 ng/L, while final effluent ranged between 1,045 and 7,665 ng/L. The highest antibiotic loading in the influent occurred in the month of February 2018 (12,780 ng/L), closely followed by December 2017 (12,584 ng/L). The highest antibiotic loading in the final effluent occurred in December 2017 (7,665 ng/L), corresponding to a removal efficiency of approximately 39%. Total antibiotic concentration between the two stages of treatment were significantly different (Wilcoxon, paired; *p* = 0.0078), exhibiting an approximate 40–50% removal efficiency.

Sulfamethoxazole and acetylsulfamethoxazole were the only sulfonamides detected among the influent and effluent samples, however, the concentration of sulfamethoxazole was never greater than the proposed no effect concentration (PNEC) (Bengtsson-Palme and Larsson, 2016), below which no selection of antibiotic resistance bacteria is anticipated (Supplementary Table 8). The concentration of acetylsulfamethoxazole was greater than sulfamethoxazole in all sampling events, except August 2018, and the two were not strongly correlated (*R* = 0.1557, *p* > 0.05). Aggregate sulfonamide antibiotics and sulfonamide ARGs were not correlated (*R* = 0.14, Bonferroni-corrected *p* > 0.05). Sulfonamides and trimethoprim antibiotics showed a strong correlation in the influent (*R* = 0.95, Bonferroni-corrected *p* = 0.0021). Trimethoprim exceeded the PNEC in seven out of eight sampling events (Supplementary Table 8), although of the ARGs detected within the trimethoprim resistant dihydrofolate reductase (dfr) gene family, only two ARGs correlated in the influent at *R* > 0.5, although the associated Bonferroni-corrected *p*-values were > 0.05. All Spearman rank correlation coefficients and p-values are available in Supplementary Data 5.

Of the MLS antibiotics, azithromycin was the most persistent from influent to final effluent, furthermore, azithromycin and clarithromycin appeared to be somewhat negatively correlated (*R* = −0.69, Bonferroni-corrected *p* > 0.05). In five out of eight sampling events, azithromycin or clarithromycin were not detected simultaneously. Anhydro erythromycin and respective MLS ARGs did not correlate, while erythromycin was not detected. Aggregate values of MLS ARGs and MLS antibiotics were not strongly correlated (*R* = 0.52, Bonferroni-corrected *p* > 0.05).

There were no strong correlations of ciprofloxacin, the only quinolone antibiotic detected of those screened, with individual or aggregate quinolone ARGs in the influent (Bonferroni-corrected *p* > 0.05). This was the case even though the concentration of ciprofloxacin exceeded the PNEC in all sampling events in which it was detected (Supplementary Table 8).

### Percent Resistance to Antibiotics Among Regional Clinical Isolates

Given that sulfamethoxazole/trimethoprim, ciprofloxacin, and macrolides (anhydro-erythromycin, azithromycin, and clarithromycin) were readily detectable in the wastewater samples, resistances to these classes of antibiotics were further examined in the available regional clinical resistance data (Virginia Department of Health, 2017). As a percentage of isolates across multiple medical facilities in the near-southwest region of Virginia, *Acinetobacter baumannii* (36% of 239 isolates), *Enterobacter cloacae* (15% of 454), *Escherichia coli* (23% of 23,761 isolates), *Klebsiella pneumoniae* (11% of 3,759 isolates) and *Staphylococcus aureus* (4% of 7,455 isolates) conferred resistance to sulfamethoxazole/trimethoprim. Similar percentages were observed for resistance to ciprofloxacin: *Acinetobacter baumannii* (33% of 151 isolates), *Enterobacter cloacae* (11% of 442 isolates), *Escherichia coli* (23% of 23,387 isolates), *Klebsiella pneumoniae* (5% of 3,274 isolates), and *Pseudomonas aeruginosa* (18% of 2,245 isolates). Erythromycin was the least effective against tested isolates: *Staphylococcus aureus* (64% of 7,692 isolates), *Streptococcus pneumoniae* (46% of 232 isolates, and *Streptococcus agalactiae* (65% of 122 isolates). Although erythromycin was not directly detected, its secondary metabolite was readily detected and at levels exceeding the PNEC (Oct 2018). As expected, antibiotics being prescribed in the local clinical environment were detectable in wastewater along with gene markers of organisms resistant to those antibiotics. While selection pressure in the WWTP is a possibility, this likely indicates these antibiotics and ARGs originate from the same sources.

## Discussion

This study provides comprehensive insight into the composition and variance of the antibiotic resistome across eight sampling events conducted at a small conventional WWTP over an 18-month period. Metagenomic sequencing targeting various stages of treatment, along with core and discriminatory ARG analysis, enabled assessment of which ARGs are most effectively removed (i.e., Categories 1 and 3) versus which increase in or are unique to the effluent (Categories 2 and 4, respectively). Further, observed patterns in composition and removal of ARGs, as well as genera containing key pathogens of antibiotic resistance concern, were compared to antibiotic measurements and regional clinical resistance patterns. The approach provided insight into the efficacy of each stage of treatment for ARG removal, while also identifying indicator ARGs and other metagenomic-derived metrics (e.g., resistome risk) relevant to treatment and clinical resistance that should be considered as future candidates for metagenomic surveillance of WWTPs. Notably, strong correlations between all targeted ARGs (excluding *van*A) measured by qPCR are an encouraging indication that shotgun metagenomic sequencing yields quantitative information that can be compared within and across WWTPs with time. However, the discrepancy between *van*A measured via qPCR and metagenomics suggests that caution should be taken in recognizing that there could be biases in metagenomic sequencing. We also recognize that there are inherent limitations in the detection limit of metagenomics and note that the sequencing applied in this study was deeper than most prior WWTP resistome studies. Finally, given that the number of features detected by metagenomics will always be greater than the sample size, there are inherent challenges in making statistical inferences (Jonsson et al., 2016; Bengtsson-Palme et al., 2017).

About 50% reduction in total ARG relative abundance was observed from influent to final effluent (i.e., 2-fold difference) across all sampling events, along with a distinct shift in the resistome composition. Approximately the same magnitude of reduction in total ARG relative abundance was reported in a previous study of three Swedish WWTPs serving 150,000–800,000 people, each sampled once (Bengtsson-Palme et al., 2016). Changes in relative abundance are a useful indicator of the relative degree of selection pressure for carriage of ARGs across a microbial community, although it is important to acknowledge that unrelated factors shaping taxonomy will also affect the resistome composition. Overall, this study is consistent with others that suggest that, while some ARGs persist or increase during wastewater treatment, the general tendency is loss or reduction in ARGs through activated sludge treatment and secondary settling (Gao et al., 2012; Bengtsson-Palme et al., 2016; Lira et al., 2020). Further, it is important to consider that there is a sharp decrease in total bacterial loads from influent to effluent, which will further decrease loads of ARGs in the effluent and likely any associated risks. In the present study, bacterial abundances (16S rRNA gene numbers) decreased by 2-orders of magnitude. Bengtsson-Palme et al. (2016) noted that accounting for the removal of total bacteria resulted in a ∼50-fold decrease in absolute abundance (i.e., ARG copies/mL) of total ARGs from influent to effluent, while 2–3 log_10_ removal in absolute abundance was observed in the present study. Here we find that both relative abundance and absolute abundance estimations are useful, with the latter considered to be more informative for risk assessment.

The number of ARGs (i.e., ARG diversity) detected from influent to secondary effluent was observed to decrease across all sampling events, from 859 to 637 (a loss in detection of 25% of ARGs). Lira et al. (2020) examined the metagenome at a 9.5 MGD conventional WWTP in Portugal over three sampling events and observed a much sharper loss in detection of ARG types from influent to final effluent (post-UV disinfection) of about 75% of ARGs, although only 259 ARGs were detected across the study. This lower detection rate is likely due to the much shallower sequencing depth obtained via MiSeq sequencing (maximum 8.2 million reads per sample reported in the Lira study, versus 37 million average reads per sample in present study) and higher stringency applied in ARG annotation (95% identity, versus 80% identity). While general trends in ARG removal determined via metagenomics were consistent across the present study and others, this highlights that differences in sequencing approach (e.g., platform, library prep, sequencing depth, sequence length) and analysis approach (e.g., databases, search criteria) will influence precisely which ARGs are detected and could lead to false positives or false negatives, especially for less abundant ARGs.

Based on this study, multidrug ARGs were found to be the most persistent, accounting for up to 40% of total ARG relative abundance in the secondary effluent. The most substantial reduction occurred in the MLS resistance class. Aminocoumarin, glycopeptide, phenicol, rifamycin, sulfonamide, trimethoprim, and other resistance classes experienced an overall increase in ARG relative abundance in at least four sampling events. February 2018 exhibited the greatest number of individual core ARGs that increased from influent to effluent (Category 2 ARGs), while June 2018 accounted for the least reduction in total ARG relative abundance.

The discriminatory resistome was comprised of ARGs that most effectively distinguished the secondary effluent from the influent. Important to note is that the discriminatory ARGs identified by the ExtrARG randomized tree algorithm (Gupta et al., 2019) are distinct from those identified based on the above comparisons of ARG detections in the secondary effluent versus influent. For example, there were several abundant core ARGs that appeared in the discriminatory resistome due to the magnitude of change in relative abundance from influent to secondary effluent. These shared ARGs included: *mph*D, *msr*E, *tet*39, *tet*Q, *ade*J, *ade*K, of which all but *tet*Q decreased in relative abundance during each sampling event (the relative abundance of *tet*Q increased in June 2018). Such genes could be monitored in future to verify consistent removal of these ARGs. Further, clinically relevant ARGs of concern that are also discriminatory could also be prioritized, including multiple OXA beta-lactamases, as well as the MLS ARGs *mph*D and *msr*E.

The relatively tight clustering of influent samples, visualized by NMDS analysis, indicated that the incoming resistome was relatively stable across the 18-month study period. Greater variability was observed in the resistome of the secondary effluent, which was further indicated by the higher COVs for each antibiotic resistance class. These results suggest that the sewage resistome of a given community served by a WWTP is relatively stable compared to that of the effluent, which is influenced by day-to-day treatment variability. Furthermore, this finding suggests locational reproducibility, and thus relatively infrequent influent sampling may be sufficient for the purpose of comparison of global trends across WWTPs. For the two sampling events when activated sludge was sequenced in this study (February 2018 and August 2018), it was noted that there was a sharp decrease in subsequent total ARG relative abundance, emphasizing the efficacy of this biological treatment stage for ARG attenuation. Still, although it was difficult to link precise changes in the influent to precise shifts in the effluent, it is important to note that ∼90% of secondary effluent ARGs were also found in the influent. The effluent also largely reflected the activated sludge, suggesting that it is heavily influenced by microbes escaping settling. The finding demonstrates that the activated sludge barrier does not completely erase the influence of the influent on the effluent resistome. This suggests that policies aimed at restricting what is discharged to WWTPs could in fact influence input of ARGs to receiving environments. Future studies aimed at assessing how influent resistomes shape effluent resistomes would be of interest.

Considering the results in terms of clinical resistance information helps to further refine potential metagenomic monitoring approaches. For example, ARGs of known clinical concern that are also found in the core and discriminatory resistomes, primarily OXA beta-lactamases, could be prioritized for monitoring and compared against the typical trends denoted by Categories 1–4 as an indicator of potential local resistance concerns and overall WWTP performance. Further, it was quite remarkable that the ARG-pathogen linkages noted in the assembled effluent metagenomes were more similar to regional patterns of clinical resistance than those found in the influent. This was especially the case in June 2018 and August 2018, both summer sampling events, with the most occurrences of increased abundance of pathogen-containing genera of local clinical importance. The tendency of achieving greater percent assembly of secondary effluent metagenomes could have attributed to higher frequency of identification of co-occurring ARG, MGE, and pathogen-like annotations than in the influent. We also acknowledge that MGE annotation is challenging due to out-of-date, incomplete, and disparate public databases, thus improving the accuracy of databases used for annotation should be prioritized in future research. Regardless, these results highlight that WWTP effluent monitoring also has the potential to shed light on local clinical antibiotic resistance concerns, as has been recently proposed with respect to monitoring sewage influents (Aarestrup and Woolhouse, 2020). Effluent monitoring also presents the advantage that it can aid in assessing WWTP performance and provides a measure of loading to the environment and potential associated exposures. Given that the variability of the effluent was higher than the influent, this suggests that it may also be possible to better detect and act upon anomalies of concern than by influent monitoring alone.

In terms of a comprehensive indicator of the potential for ARGs to spread and be present in human pathogens, MetaCompare showed promise in this study. In particular, MetaCompare analysis provided a comprehensive metric that takes into account ARGs, their mobility, and potential presence in pathogens. In this way, relative comparisons of resistome risk can be made in time or space for a given system to prioritize further investigation or action. Consistent with reduction of pathogens from influent, resistome risk scores also consistently decreased through the WWTP in this study. Still, taxonomic markers corresponding to the genus-level of all nine pathogens monitored in the local clinical antibiotic surveillance could be detected in the effluent and sometimes even increased in relative abundance. *Escherichia* stood out in this study both as having the most ARG connections on assembled contigs in common with local antibiogram data and tending to increase in relative abundance in the effluent. Extended-spectrum beta-lactamase producing *E. coli* are regularly isolated from treated wastewater effluent globally and are being considered by the World Health Organization (2021) and others as a standardized monitoring target for antibiotic resistance in wastewater (Diallo et al., 2013; Bréchet et al., 2014; Li et al., 2019; Marano et al., 2020).

There did not appear to be distinct grouping of influent resistomes by season (winter, spring, summer, fall) or grouped seasons (winter/spring, summer/fall) based on ARG profiles. This finding is consistent with no observed seasonality in the monthly sampling of activated sludge at a Hong Kong WWTP over 9 years, although the authors hypothesized that this was due to minimal local shifts in temperature (Yin et al., 2019). The distinct separation of influent from activated sludge, secondary effluent, and final effluent in the NMDS plots is consistent with the trends in mobile ARGs and plasmid replicons observed among three sampling events over 6 months at the Portuguese WWTP noted above (Lira et al., 2020), consistent with the above observation of relatively stable resistome in the influent that subsequently shifts and increases in variability in the effluent. Interestingly, it was noted that the activated sludge resistome shifted in composition every 2–3 years at the Hong Kong WWTP (Yin et al., 2019), a turnover period that would not have been captured over the duration of the present study. Still, the fact that the effluent resistome was generally more variable over the period of this study than the influent resistome suggests that there may have been some variability in the activated sludge resistome of the prior treatment stage as well (although it was only sequenced on two sampling dates).

While the microbiome was also distinct in composition for each stage of treatment, its stability mirrored that of the resistome throughout the 18-month sampling period. The dominance of *Arcobacter* and *Acinetobacter* in the influent was not unlike a previous metagenomic study comparing the resistome and microbiome of coastal beach versus sewage waters in Uruguay (Fresia et al., 2019). In contrast, *Pseudomonas* was the most abundant genera in the sewage waters in this study, whereas *Pseudomonas* was one of the lesser dominant genera in the top 20 most abundant. *Enhydrobacter* was not a discussed in the Uruguayan study. Although the relative abundances of *Arcobacter* and *Acinetobacter* decreased from the influent through activated sludge, their average abundances remained similar to the core OTUs found in activated sludge compartments globally (Wu et al., 2019): *Arcobacter* (0.28%, versus 0.43% in global study) and *Acinetobacter* (1.07%, versus 0.22% in global study). Of the more dominant genera found from activated sludge onward, only *Nitrospira* was present as a core global OTU (Wu et al., 2019). Fresia et al. (2019) also utilized MetaPhlAn2 for taxonomic annotation, while Wu et al. (2019) relied on 16S rRNA amplicon sequencing.

Total measured antibiotic load also decreased by ∼40–50%, by the same order of magnitude of total ARG relative abundance. Otherwise, the fate of antibiotics was not predictive of the fate of respective ARGs. For example, MLS antibiotics, especially azithromycin, were the most persistent among the measured antibiotic classes and sometimes even increased in concentration from influent to final effluent. MLS ARGs, on the other hand, consistently decreased from influent to secondary effluent. Even for antibiotic concentrations greater than the PNEC, in most cases there were not significant correlations with ARGs. For example, the concentration of ciprofloxacin was greater than the PNEC in seven sampling events, yet no significant correlations with corresponding quinolone ARGs were observed. We acknowledge that difficulty detecting clinically relevant quinolone ARGs, due to exclusion of resistance due to point mutation (which is often the case for clinically relevant quinolone ARGs) in the metagenomic analysis, could have influenced this analysis. One limitation of the current study is difficulty in detecting beta-lactams, which represented a large portion of the discriminatory resistome and Category 4 ARGs. However, beta-lactams degrade rapidly under environmental conditions and few studies have reported their detection in WWTPs (Singh et al., 2019). Generally, other studies have similarly noted lack of correlation between antibiotic residues and ARG abundance (Bengtsson-Palme et al., 2016; Hendriksen et al., 2019; Pärnänen et al., 2019; Riquelme et al., in preparation). Thus, growing consensus among several studies supports the overall conclusion that selection pressures for carriage of ARGs across the microbial community are generally diminished through the WWTP. This is consistent with the reduced total ARG relative abundance from influent to effluent consistently observed across this study. Still, monitoring antibiotics can be informative to gain insight into which antibiotics are being used in a given community, which is difficult to ascertain from publicly available data (Morgan et al., 2011; Auta et al., 2019), is also important surveillance information.

## Conclusion

Based on this intense 18-month study of a conventional WWTP and comparison to locally available clinical resistance information, metagenomic analysis was found to yield rich information about resistomes and associated microbiomes that can be mined to inform effective strategies for antibiotic resistance surveillance. In terms of metrics that are worthy of consideration for future monitoring efforts, both core and discriminatory resistome analysis revealed several ARGs of clinical concern, while MetaCompare analysis provided a comprehensive metric for relative comparison of the degree to which ARGs are predicted to be mobile and carried in pathogens (i.e., resistome risk).

Overall, it was observed that most antibiotic resistance indicators of concern decreased during wastewater treatment, including: measured antibiotics (although some macrolides increased), relative abundance of total ARGs, absolute abundance of total ARGs, and resistome risk. Together with Category 1 and Category 3 ARGs, which are either completely removed or decrease during treatment, such metrics could routinely be monitored to verify that a given WWTP is performing according to baseline. On the other hand, monitoring Category 2 ARGs, which tend to increase during treatment, could be informative for optimizing WWTP for maximal attenuation of ARGs. Further, this study provided insight into the behavior of specific ARGs of clinical relevance. Clinically relevant ARGs that are also discriminatory (e.g., *bla*OXA) could be prioritized for surveillance, either through metagenomics or qPCR-based methods.

Remarkably, it was observed that linked ARG-MGE-taxonomic information found on assembled contigs in the WWTP effluent were more similar to antibiotic resistant pathogens identified to be of local concern, relative to those found on contigs from the influent sewage. These results suggest that monitoring WWTP effluent can also provide valuable surveillance information, as has recently been proposed for WWTP influents. The fact that effluent resistomes were also more variable than influent resistomes further suggests that more frequent monitoring of effluents may be warranted than for influents and could serve to identify important upsets or anomaly events. Effluent is also valuable to monitor given that it also represents the worst case of what can be discharged to the environment and result in exposure, either through recreation in receiving waters or water reuse. Based on the findings of this study, we emphasize the following specific considerations for metagenomic-based surveillance of antibiotic resistance in WWTPs:

• Metagenomic-based surveillance of antibiotic resistance can be economized by less frequent sampling of sewage influents if the purpose is global comparison.
• Metagenomic surveillance of effluents is a promising approach to assessing both efficacy of WWTP for mitigation, but also for assessing the local clinical antibiotic resistance condition.

• Core and discriminatory resistomes can be compared within and among WWTPs with time to identify similarities and differences. About 50% reduction in relative abundance of total ARGs was achieved across the WWTP and several specific ARG types were consistently observed to be removed or to increase. Diversity of ARGs consistently decreased. Such measures could be used to benchmark WWTP performance and assess potential operational deficiencies or upsets.
• Core and discriminatory ARG analysis comparing influent and secondary effluent identified a collection of “indicator” ARGs that are also clinically relevant; such as *bla*OXA, *bla*GES, *mph*D, *msr*E, and *qnr*S, that should be further considered as targets for antibiotic resistance monitoring in WWTPs.
• Metagenomic analysis of co-occurrences of ARGs, MGEs, and pathogen gene markers was notably consistent with patterns in resistance of locally available *E. coli* monitoring data, suggesting value in coordinating with recent initiatives to target *E. coli* for antibiotic resistance surveillance of water environments. *S. aureus* harboring MLS ARGs also emerged as a target worth exploring in future research.
• While antibiotics generally tended to decrease across the WWTP, they were not generally predictive of patterns observed in corresponding ARG increase or removal.

## Data Availability Statement

The datasets generated for this study can be found in the NCBI Short Read Archive (SRA) under the BioProject ID PRJNA683044.

## Author Contributions

HM led the writing of this manuscript, along with AP, MR, BD, and PV. HM, MR, PV, and AP contributed to the experimental design. HM, AP, BD, SG, and EG contributed to the rationale of the metagenomic analysis approach. HM, BD, and SG conducted sampling at the WWTP. DA and LA carried out the antibiotic analysis. All authors read and commented on the manuscript.

## Conflict of Interest

MR was employed by VirginiaTech during the majority of study, but her affiliation switched to Diversigen, Inc., from the time of DNA sequencing and thereafter. MR did not conduct any data analyses for this manuscript. The remaining authors declare that the research was conducted in the absence of any commercial or financial relationships that could be construed as a potential conflict of interest.
