# Supplementary material for: Evaluation of Metagenomic-Enabled Antibiotic Resistance Surveillance at a Conventional Wastewater Treatment Plant
Source: Front Microbiol. 2021 May 13;12:657954. doi: 10.3389/fmicb.2021.657954 (PMC8155483; doi:10.3389/fmicb.2021.657954)
Supplement: Supplementary file 2 [file Data_Sheet_1.PDF]

Supplementary Figures and Tables

Evaluation of metagenomic-enabled antibiotic resistance surveillance at a conventional wastewater treatment plant

Haniyyah J. Majeed[1], Maria Virginia Riquelme[1,2], Benjamin C. Davis[1], Suraj Gupta[3], Luisa Angeles[4], Diana S. Aga[4], Emily Garner[1,5], Amy Pruden[1]*, and Peter J. Vikesland[1]*

[1] Virginia Polytechnic Institute and State University, Department of Civil & Environmental Engineering, Blacksburg, VA, United States

[2] Current Affiliation: Diversigen, Inc., Houston, TX, United States

[3] Virginia Polytechnic Institute and State University, Interdisciplinary PhD Program in Genetics, Bioinformatics, and Computational Biology, Blacksburg, VA, United States

[4] University at Buffalo, Department of Chemistry, Buffalo, NY, United States

[5] Current Affiliation: West Virginia University, Department of Civil & Environmental Engineering, Morgantown, WV, United States

*** Correspondence:**
Amy Pruden
apruden@vt.edu

Peter Vikesland
pvikes@vt.edu

Supplementary Materials and Methods

Manual curation of the Comprehensive Antibiotic Resistance Database

The purpose of manual curation of the Comprehensive Antibiotic Resistance Database (CARD) database was to modify the classification of resistance genes based on the literature associated with each gene. For example, the CARD database may list an ARG as being resistant to “monobactam, cephalosporin, penam,” whereby we classified it as “beta-lactam” based on the broader antibiotic class. The only gene removed from the analysis was Nocardia rifampin resistant beta-subunit of RNA polymerase (*rpo*B2).

Supplementary Data

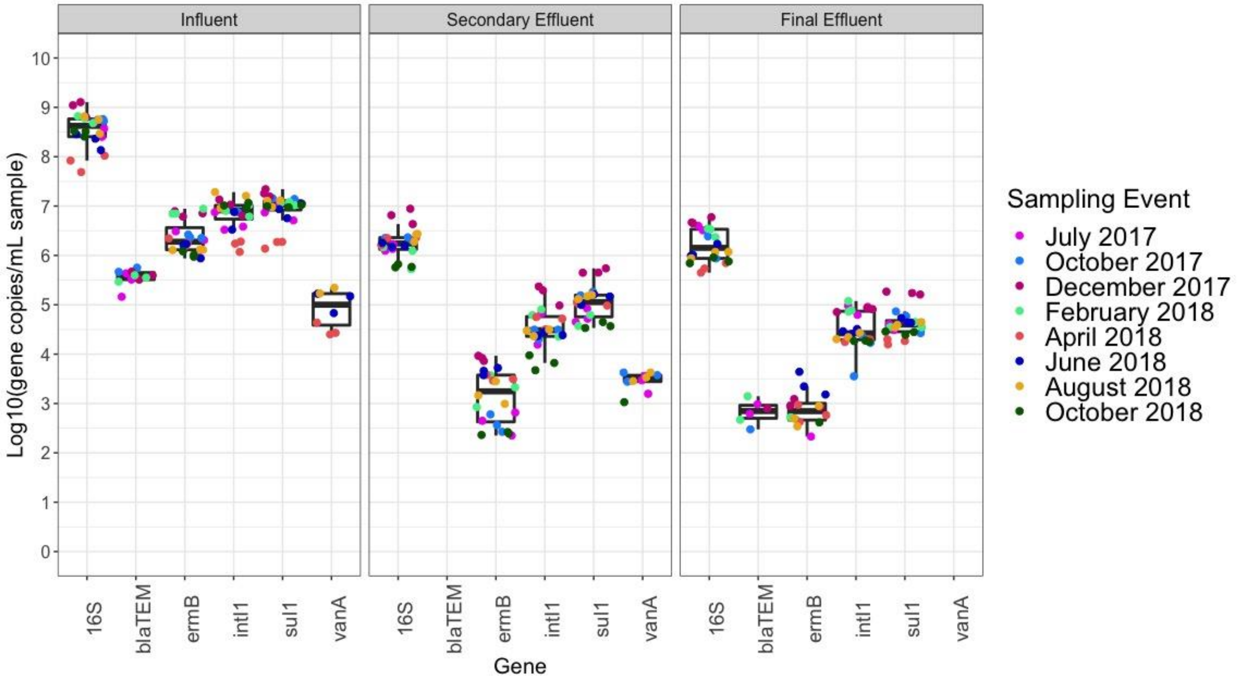

Supplementary Figure 1. Absolute abundance (as log10 gene copies per mL of sample) of select ARGs, a class 1 integron integrase gene, and total bacterial 16S rRNA genes in the influent, secondary effluent, and final effluent measured by qPCR. Primers, R^2 values, and efficiencies of the standard curves of each assay are reported in Supplementary Table 9.

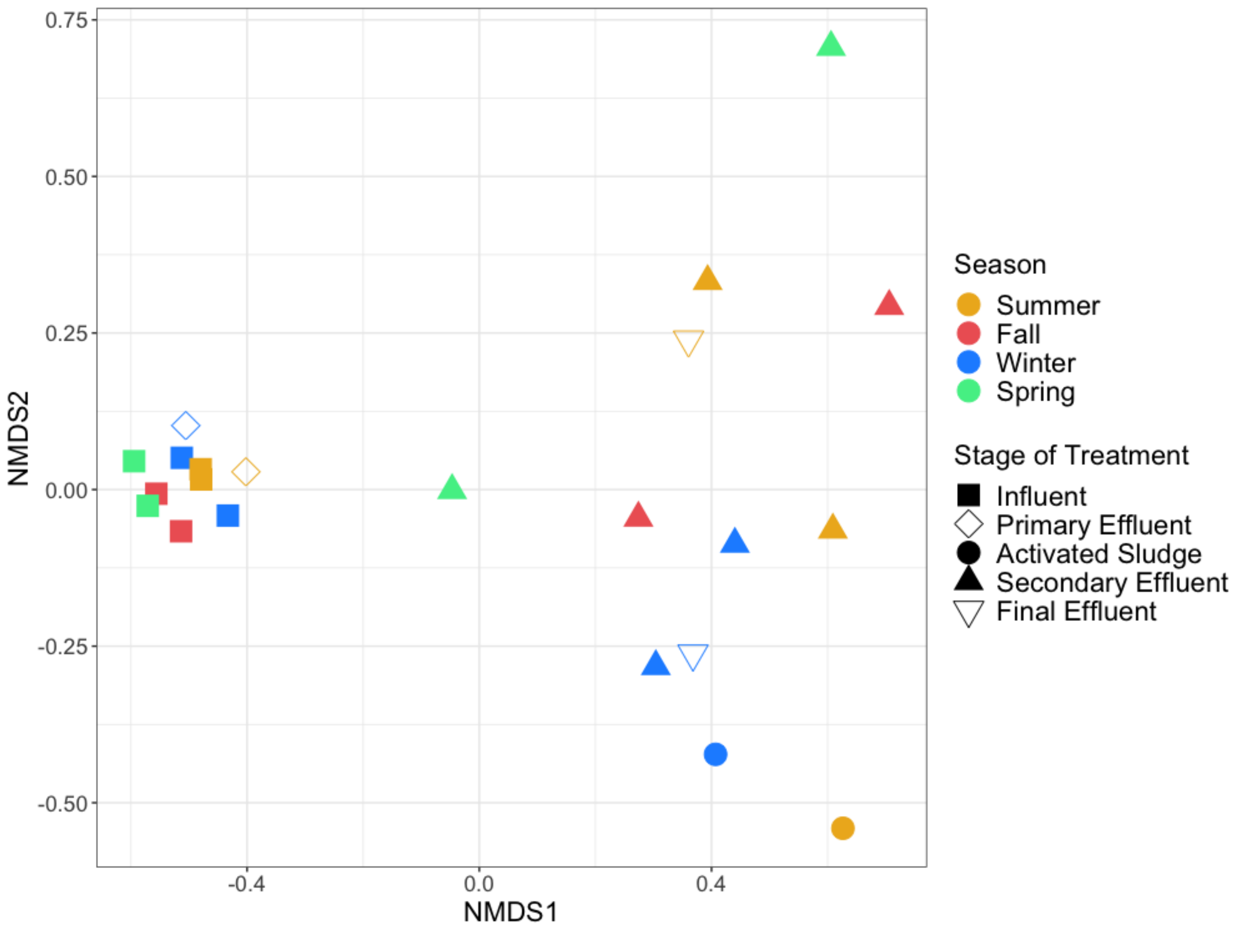

Supplementary Figure 2. NMDS analysis of ARG profiles based on relative abundance across WWTP sampling locations and seasons according to shotgun metagenomic sequencing. Influent samples did not exhibit distinct separation based on relative abundance of ARGs when grouped by season (ANOSIM; #ARGS = 859, R = 0.1458, p = 0.241), likewise secondary effluent samples were not separated by season (ANOSIM; #ARGS = 637, n = 2, R = -0.1667, p = 0.785). ARGs were annotated via CARD (Jia et al., 2017).

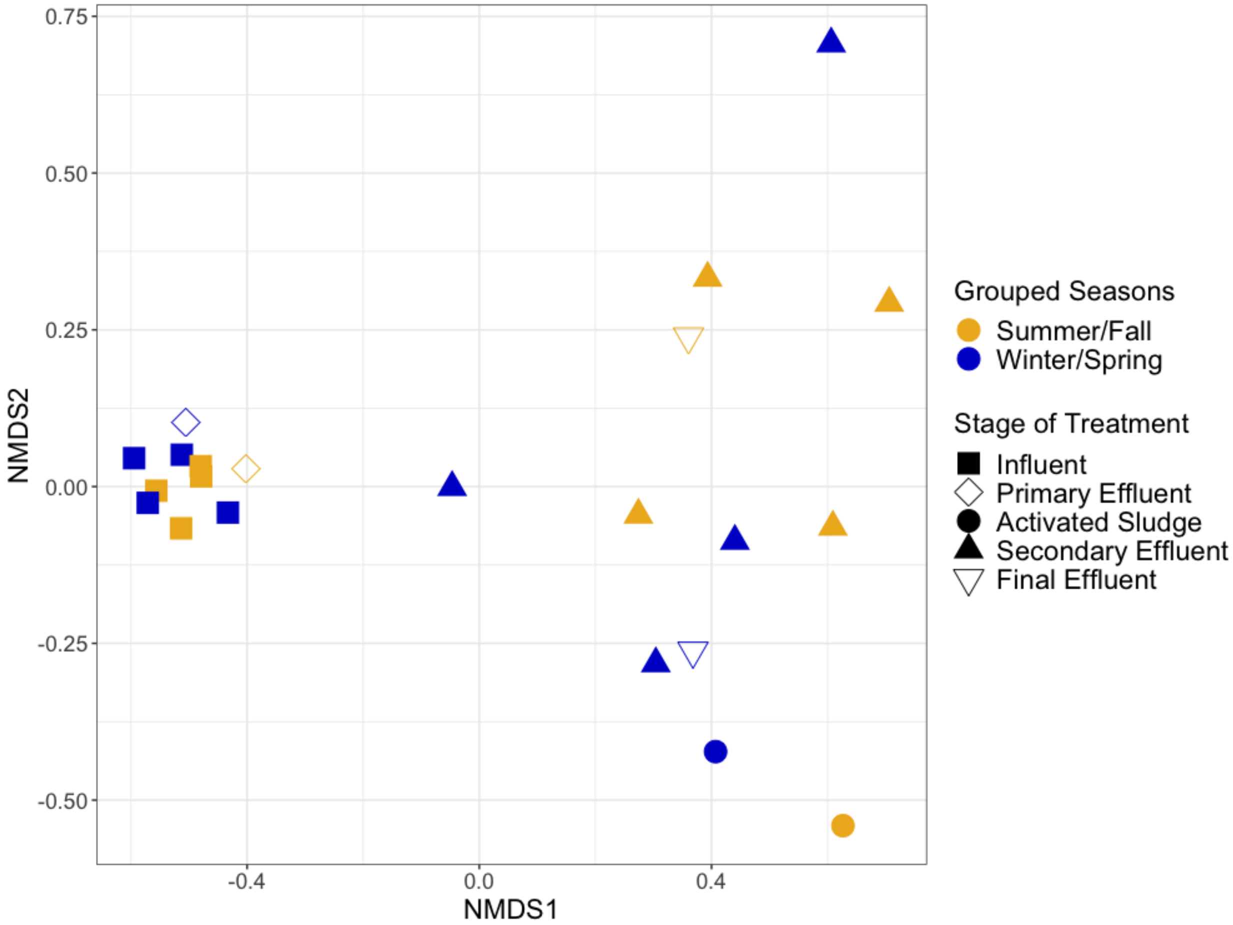

Supplementary Figure 3. NMDS analysis of ARG profiles based on relative abundance across WWTP sampling locations and grouped seasons according to shotgun metagenomic sequencing. Influent samples did not exhibit distinct separation based on relative abundance of ARGs based on grouped seasons (ANOSIM; #ARGS = 859, $R = 0.2396$, $p = 0.064$), likewise secondary effluent samples were not separated by grouped seasons (ANOSIM; #ARGS = 637, n = 4, $R = -0.04167$, $p = 0.63$). ARGs were annotated via CARD (Jia et al., 2017).

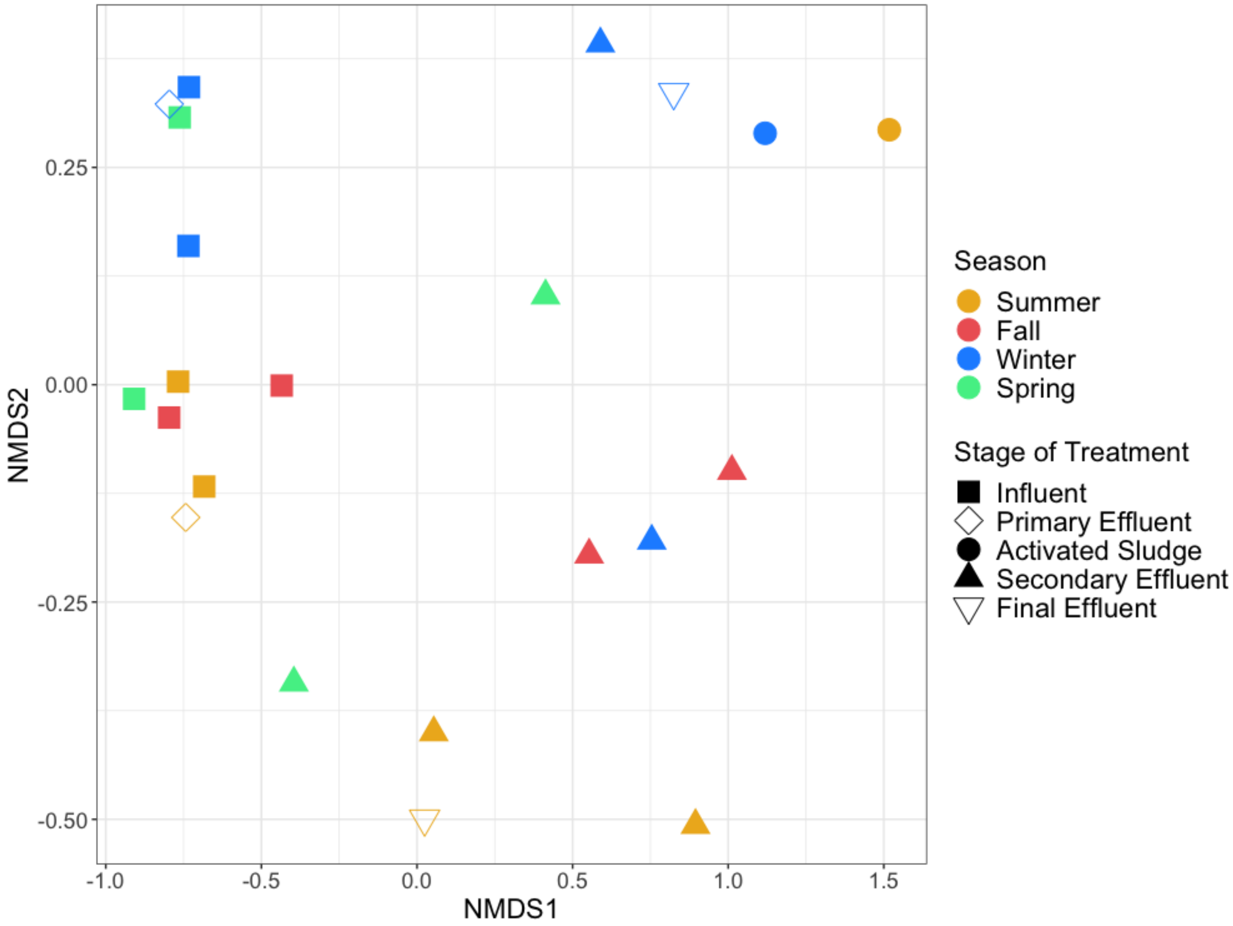

Supplementary Figure 4. NMDS analysis of taxonomic profiles at genus level across WWTP sampling locations and seasons according to shotgun metagenomic sequencing. Influent samples did not exhibit distinct separation based on genus level relative abundance by season (ANOSIM; $R = 0.5208$, $p = 0.04$), likewise secondary effluent samples were not separated by season (ANOSIM; $R = -0.1458$, $p = 0.775$). Taxonomy was annotated via MetaPhlAn2 (Truong et al., 2015).

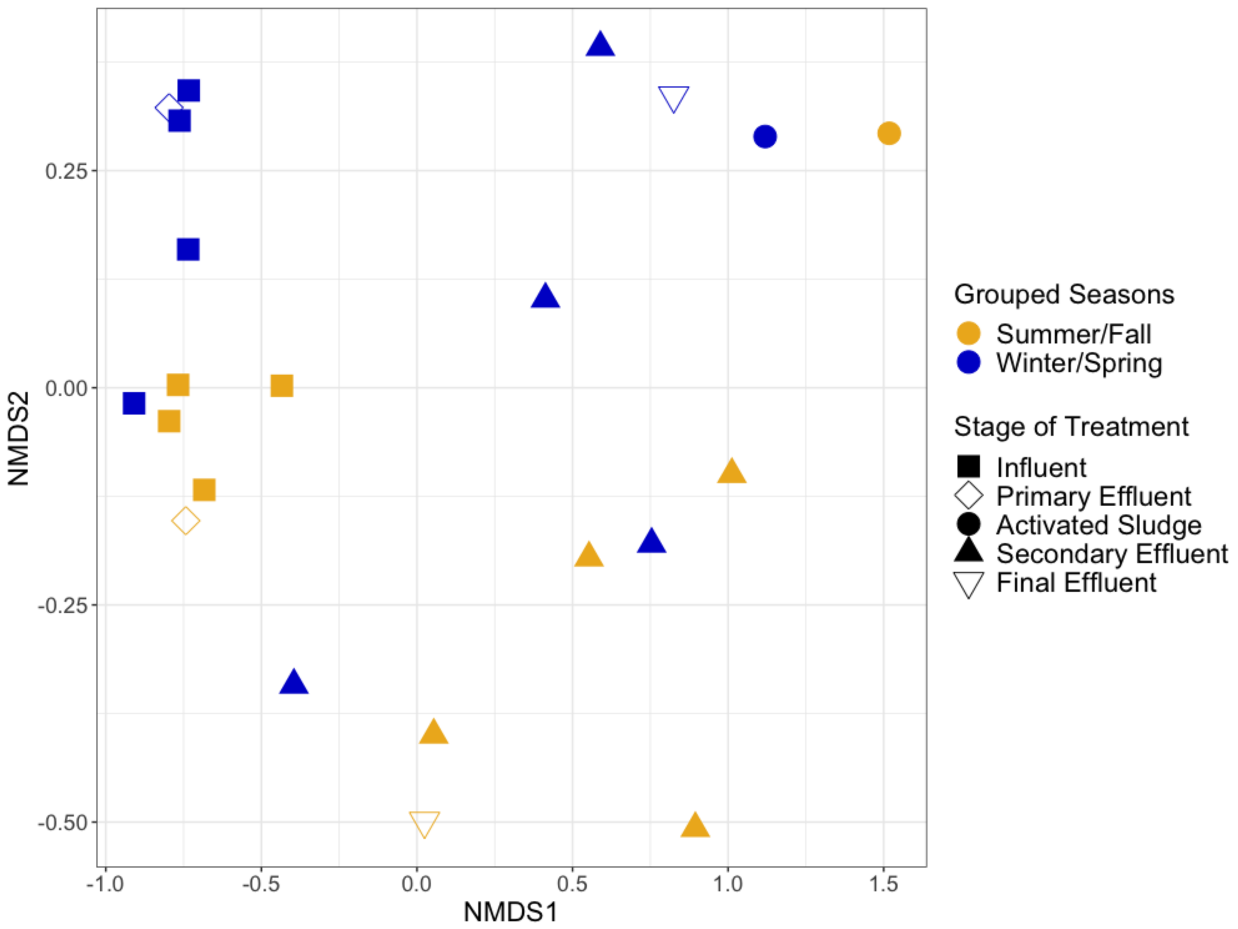

Supplementary Figure 5. NMDS analysis of taxonomic profiles at genus level across WWTP sampling locations and grouped seasons according to shotgun metagenomic sequencing. Influent samples did not exhibit distinct separation based on genus level relative abundance by grouped seasons (ANOSIM; $R = 0.1354$, $p = 0.213$), likewise secondary effluent samples were not separated by grouped seasons (ANOSIM; $R = -0.1146$, $p = 0.789$). Taxonomy was annotated via MetaPhlAn2 (Truong et al., 2015).

Supplementary Table 1. Sampling data and notes corresponding to each sampling event

Date	Ambient Temperature (°C)	Stage of Treatment	Temperature of Sample (°C)	Dissolved Oxygen (mg/L)	pH	TSS (mg/L)[a,b]	BOD (mg/L)[a,b]
**2017-07-19**	22[c]	Influent	20.6	3.31	6.94	215	-
		Secondary Effluent	23.1	2.06	6.27	5.9, 2.5	3.8, 3.7
**2017-10-17**	13	Influent	19.8	3.18	6.95	220	-
		Secondary Effluent	20.1	3.63	6.56	4.4, 8.9	
**2017-12-14**	3[c]	Influent	14.1	5.65	7.7	200	-
		Secondary Effluent	12.7	2.4	6	10.1, 10.7	10.8, 10
**2018-02-21**	19 [c]	Influent	11.8	7.05	7.65	215	-
		Primary Effluent	11.8	5.08	6.96	-	-
		Activated Sludge	12.7	0.43	6.27	-	-
		Secondary Effluent	12.7	3.6	6.32	7.1, 4.7	6.1, 4.7
		Final Effluent	13.4	8.1[d]	6.5	7.1, 4.7	6.1, 4.7
**2018-04-27**	16[c]	Influent	14	8.18	7.28	160	-
		Secondary Effluent	14.8	6.48	4.48	6.6, 6.7	4.3, 6.9
**2018-06-26**	21[c]	Influent	20	4.91	7.18	215	-
		Secondary Effluent	20.8	3.37	6.71	3.9	2.8
**2018-08-13**	24	Influent	22	2.68	7.06	160	-
		Primary Effluent	22	0.75	6.66	-	-
		Activated Sludge	22.8	0.35	6.31	-	-
		Secondary Effluent	23.2	2.82	6.53	4.8, 4.9	4.6, 3.9
		Final Effluent	22.5	6.42	6.59	4.8, 4.9	4.6, 3.9
**2018-10-24**	11	Influent	18.6	3.73	7.26	200	-
		Secondary Effluent	18	1.58	6.35	6.8, 5.6	4.2, 4.3

[a]TSS and BOD are measured in the final effluent approximately weekly at wastewater treatment facility. Final effluent measurements will be taken as secondary effluent measurements in this study.
[b]Weekly values provided before and after the sampling event, unless sampling coincided with weekly data point.
[c]Temperature taken from archived weather data available at time of sampling.

[d]Measurement taken from plant data.
[e]No recycling of activated sludge occurring due to high flow conditions caused by heavy periods of rainfall. No nitrification occurring.

Supplementary Table 2. Raw read and post-quality control (QC) sequence metrics performed via MetaStorm (Arango-Argoty et al., 2016).

Sample_ID	Stage of Treatment	Raw Read Pairs	Read Pairs After QC	%Reads Pairs Passing QC
17-DEC-IN	Influent	37366657	36449253	97.54
17-DEC-SE	Secondary Effluent	37038559	36069946	97.38
17-JUL-IN	Influent	35676038	34847071	97.68
17-JUL-SE	Secondary Effluent	38373440	37342749	97.31
17-OCT-IN	Influent	43579744	42695000	97.97
17-OCT-SE	Secondary Effluent	44250971	43125445	97.46
18-APR-IN	Influent	26192397	25628288	97.85
18-APR-SE	Secondary Effluent	40754048	39785292	97.62
18-AUG-AS	Activated Sludge	29835249	29140007	97.67
18-AUG-FE	Final Effluent	44511900	43285007	97.24
18-AUG-IN	Influent	28703438	28006923	97.57
18-AUG-PE	Primary Effluent	54498571	53259913	97.73
18-AUG-SE	Secondary Effluent	35155619	34313756	97.61
18-FEB-AS	Activated Sludge	45702582	44316672	96.97
18-FEB-FE	Final Effluent	34810818	33948558	97.52
18-FEB-IN	Influent	43601844	42551990	97.59
18-FEB-PE	Primary Effluent	29338422	28671670	97.73
18-FEB-SE	Secondary Effluent	42808929	41843448	97.74
18-JUN-IN	Influent	46660742	45691625	97.92
18-JUN-SE	Secondary Effluent	41261213	40328173	97.74
18-OCT-IN	Influent	3736000	3651048	97.73
18-OCT-SE	Secondary Effluent	37820256	36922790	97.63

Supplementary Table 3. List of 61 category 3 ARGs (ARGs which decreased in the secondary effluent with respect to the influent across all sampling events).

**ARGs**
*aad*A2
*aad*A8
*abe*M
*acr*B
*acr*D
*acr*F
*ade*B
*ade*I
*ade*J
*ade*K
*bla*AER-1
*bla*OXA-16
aph(3")-Ib
aph(6)-Id
*arn*A
*bac*A
*bae*R
*bae*S
*cml*A5
*cpx*A
crp
*dfr*A3
*emr*A
*emr*B
*emr*D
*acr*A in *Enterobacter cloacae*
*erm*B
lamb in *Escherichia coli*
*mdf*A in *Escherichia coli*
*gol*S
*omp*K35 in *Klebsiella pneumoniae*
*omp*K36 in *Klebsiella pneumoniae*
*mds*B
*mds*C
*mdt*A
*mdt*H
*mef*C
mel
*mex*A
*mex*L
*mph*D
*mph*G
*msr*E
*mva*T
*opm*H
*opr*J

Supplementary Table 4. ANOSIM pairwise tests for relative abundance of all antibiotic resistance genes across all treatment processes.

Groups (n[a])	R-statistic	Significance level, *p*
**Influent (n=8), Secondary Effluent (n=8)**	0.933	0.001
**Influent (n=8), Activated Sludge (n=2)**	1	0.022
**Influent (n=8), Final Effluent (n=2)**	1	0.022
**Influent (n=8), Primary Effluent (n=2)**	0.228	0.178
**Secondary Effluent (n=8), Activated Sludge (n=2)**	0.151	0.267
**Secondary Effluent (n=8), Final Effluent (n=2)**	-0.306	0.911
**Secondary Effluent (n=8), Primary Effluent (n=2)**	0.776	0.022
**Activated Sludge (n=2), Final Effluent (n=2)**	0.25	0.333
**Activated Sludge (n=2), Primary Effluent (n=2)**	1	0.333
**Final Effluent (n=2), Primary Effluent (n=2)**	1	0.333

[a]n = number of samples within group

Supplementary Table 5. ANOSIM pairwise tests for all genus level relative abundance across all treatment processes.

Groups (n[a])	R-statistic	Significance level, *p*
**Influent (n=8), Secondary Effluent (n=8)**	0.801	0.002
**Influent (n=8), Activated Sludge (n=2)**	1	0.019
**Influent (n=8), Final Effluent (n=2)**	1	0.024
**Influent (n=8), Primary Effluent (n=2)**	0.31	0.136
**Secondary Effluent (n=8), Activated Sludge (n=2)**	0.289	0.15
**Secondary Effluent (n=8), Final Effluent (n=2)**	-0.172	0.795
**Secondary Effluent (n=8), Primary Effluent (n=2)**	0.547	0.03
**Activated Sludge (n=2), Final Effluent (n=2)**	0.25	0.667
**Activated Sludge (n=2), Primary Effluent (n=2)**	1	0.333
**Final Effluent (n=2), Primary Effluent (n=2)**	0.75	0.333

[a]n = number of samples within group

Supplementary Table 6. Assembly information presented in MetaStorm (Arango-Argoty et al., 2016). Values determined to calculate relative risk scores using MetaCompare (Oh et al, 2018).

Sample	Percent Assembled	#Contigs	#ARG	#MGE	#PAT	Q(ARG)	Q(ARG_MGE)	Q(ARG_MGE _PAT)	Risk Score
**17-DEC-IN**	43	654151	3594	8295	13043	0.00549414	0.00023542	0.00006268	36.46
**17-DEC-SE**	54	587366	849	4993	501	0.00144544	0.00010896	0.00001192	21.37
**17-JUL-IN**	41	630444	3210	7458	12498	0.00509165	0.00023317	0.00007138	34.89
**17-JUL-SE**	69	315513	540	2804	362	0.0017115	0.0001553	0.00000951	22.26
**17-OCT-IN**	43	749710	4118	9332	20201	0.00549279	0.0002561	0.00008403	36.83
**17-OCT-SE**	58	525930	924	3497	714	0.00175689	0.00011408	0.00003042	22.32
**18-APR-IN**	43	463657	2425	5967	6815	0.00523016	0.00022215	0.00004961	35.18
**18-APR-SE**	57	507711	756	3697	441	0.00148904	6.89E-05	0.00000788	21.35
**18-AUG-AS**	48	478979	1032	4654	589	0.00215458	0.00010856	0.00001044	23.38
**18-AUG-FE**	51	572315	1190	4419	1226	0.00207927	0.00010833	1.92E-05	23.19
**18-AUG-IN**	39	515184	2430	5791	7360	0.00471676	0.00020963	0.00005241	33.08
**18-AUG-PE**	39	964871	3899	9467	13041	0.00404095	0.00015961	0.00004975	30.14
**18-AUG-SE**	53	479626	921	3548	916	0.00192025	9.59E-05	0.00002502	22.7
**18-FEB-AS**	59	627702	1204	7677	684	0.00191811	0.00016409	0.00001593	22.91
**18-FEB-FE**	59	446325	794	4527	504	0.00177897	0.00012099	0.00000896	22.33
**18-FEB-IN**	44	778402	3151	9131	10364	0.00404804	0.00017472	0.00003469	30.17
**18-FEB-PE**	41	523362	2467	6071	11520	0.00471375	0.00021591	0.00005923	33.16
**18-FEB-SE**	53	494224	842	5232	516	0.00170368	0.00013961	0.00001619	22.2
**18-JUN-IN**	42	819981	4211	9617	17676	0.00513548	0.00024025	0.00009147	35.29
**18-JUN-SE**	50	504902	1062	4478	1505	0.00210338	0.00019014	0.00004357	23.66
**18-OCT-IN**	34	74873	368	1265	866	0.00491499	0.00030719	8.01E-05	34.83
**18-OCT-SE**	24	14876	9	21	8	0.000605	0	0	18.92

Supplementary Table 7. Clinically-relevant ARGs in each resistome.

Resistome Type (number of clinically-relevant ARGs out of total ARGs in specific resistome)	List of clinically-relevant ARGs present
**Core of influent and secondary effluent (11/143)**	*bla*OXA-3, *bla*OXA-5, *bla*OXA-16, *bla*OXA-46, *bla*OXA-74, *bla*OXA-118, *bla*OXA-129, *bla*OXA-145, *bla*OXA-205, *bla*OXA-210, *qnr*S2
**Discriminatory (4/32)**	*bla*GES-22, *bla*OXA-212, *bla*OXA-309, *bla*OXA-333

Supplementary Table 8. Concentrations (ng/L) of antibiotics detected in influent and final effluent samples. Values in parentheses are the proposed no effect concentration (PNEC) (Bengtsson-Palme & Larsson, 2016) below which no selection of antibiotic resistance bacteria is anticipated. Bold font reflects antibiotic concentrations greater than the PNEC.

Sampling Event	Stage of Treatment	**MLS**							
		**A-ERY (1000 ng/L)**	**AZI (250 ng/L)**	**CLA (250 ng/L)**	**ERY (1000 ng/L)**	**ROX (1000 ng/L)**	**SPI I (500 ng/L)[a]**	**SPI II**	**SPI III**
July 2017	Influent	64.44	140.30	43.35	n.d.	n.d.	n.d.	n.d.	n.d.
	Final Effluent	50.27	182.09	44.33	n.d.	n.d.	n.d.	n.d.	n.d.
October 2017	Influent	339.25	**2270.39**	n.d.	n.d.	n.d.	n.d.	n.d.	n.d.
	Final Effluent	152.09	**3586.24**	n.d.	n.d.	n.d.	n.d.	n.d.	n.d.
December 2017	Influent	n.d.	**6842.01**	n.d.	n.d.	n.d.	n.d.	n.d.	n.d.
	Final Effluent	335.59	**6169.65**	n.d.	n.d.	n.d.	n.d.	n.d.	n.d.
February 2018	Influent	n.d.	202.05	155.21	n.d.	n.d.	n.d.	n.d.	n.d.
	Final Effluent	22.76	**373.49**	165.24	n.d.	n.d.	n.d.	n.d.	n.d.
April 2018	Influent	n.d.	n.d.	**694.49**	n.d.	n.d.	n.d.	n.d.	n.d.
	Final Effluent	n.d.	n.d.	**1082.52**	n.d.	n.d.	n.d.	n.d.	n.d.
June 2018	Influent	n.d.	n.d.	16.89	n.d.	n.d.	n.d.	n.d.	n.d.
	Final Effluent	55.56	**422.08**	40.48	n.d.	n.d.	n.d.	n.d.	n.d.
August 2018	Influent	n.d.	**648.73**	n.d.	n.d.	n.d.	n.d.	n.d.	n.d.
	Final Effluent	22.46	**419.75**	n.d.	n.d.	n.d.	n.d.	n.d.	n.d.
October 2018	Influent	**1564.22**	**479.63**	**425.71**	n.d.	n.d.	n.d.	n.d.	n.d.
	Final Effluent	n.d.	**469.64**	220.69	n.d.	n.d.	n.d.	n.d.	n.d.

[a]PNEC established for spiramycin, not specifically spiramycin I, II, or III.

QUINOLONE						TETRACYCLINE				OTHER
**CIP (64 ng/L)**	**ENRO (64 ng/L)**	**NOR (500 ng/L)**	**OXO**	**SARA**	**TIL (1000 ng/L)**	**ATC**	**CTC**	**OTC**	**TET (1000 ng/L)**	**TYL (4000 ng/L)**
**768.31**	n.d.	n.d.	n.d.	n.d.	n.d.	n.d.	n.d.	n.d.	n.d.	n.d.
**156.22**	n.d.	n.d.	n.d.	n.d.	n.d.	n.d.	n.d.	n.d.	n.d.	n.d.
**1688.42**	n.d.	n.d.	n.d.	n.d.	n.d.	n.d.	n.d.	n.d.	n.d.	n.d.
**161.70**	n.d.	n.d.	n.d.	n.d.	n.d.	n.d.	n.d.	n.d.	n.d.	n.d.
**2174.61**	n.d.	n.d.	n.d.	n.d.	n.d.	n.d.	n.d.	n.d.	n.d.	n.d.
**277.05**	n.d.	n.d.	n.d.	n.d.	n.d.	n.d.	n.d.	n.d.	n.d.	n.d.
**6314.85**	n.d.	n.d.	n.d.	n.d.	n.d.	n.d.	n.d.	n.d.	n.d.	n.d.
**453.28**	n.d.	n.d.	n.d.	n.d.	n.d.	n.d.	n.d.	n.d.	n.d.	n.d.
**1746.15**	n.d.	n.d.	n.d.	n.d.	n.d.	n.d.	n.d.	n.d.	n.d.	n.d.
**356.92**	n.d.	n.d.	n.d.	n.d.	n.d.	n.d.	n.d.	n.d.	n.d.	n.d.
**770.45**	n.d.	n.d.	n.d.	n.d.	n.d.	n.d.	n.d.	n.d.	n.d.	n.d.
**235.65**	n.d.	n.d.	n.d.	n.d.	n.d.	n.d.	n.d.	n.d.	n.d.	n.d.
n.d.	n.d.	n.d.	n.d.	n.d.	n.d.	n.d.	n.d.	n.d.	n.d.	n.d.
n.d.	n.d.	n.d.	n.d.	n.d.	n.d.	n.d.	n.d.	n.d.	n.d.	n.d.
**2178.37**	n.d.	n.d.	n.d.	n.d.	n.d.	n.d.	n.d.	n.d.	n.d.	n.d.
**434.52**	n.d.	n.d.	n.d.	n.d.	n.d.	n.d.	n.d.	n.d.	n.d.	n.d.

SULFONAMIDE										TRIMETHOPRIM
**A-SMX**	**SCP**	**SPD**	**SDM**	**SMR**	**SMZ**	**SMI**	**SMX (16000 ng/L)**	**SMT**	**STZ**	**TMP (500 ng/L)**
916.89	n.d.	n.d.	n.d.	n.d.	n.d.	n.d.	284.44	n.d.	n.d.	396.08
73.12	n.d.	n.d.	n.d.	n.d.	n.d.	n.d.	71.78	n.d.	n.d.	466.87
2863.64	n.d.	n.d.	n.d.	n.d.	n.d.	n.d.	n.d.	n.d.	n.d.	**994.39**
109.11	n.d.	n.d.	n.d.	n.d.	n.d.	n.d.	228.60	n.d.	n.d.	353.59
2331.33	n.d.	n.d.	n.d.	n.d.	n.d.	n.d.	697.91	n.d.	n.d.	**538.31**
n.d.	n.d.	n.d.	n.d.	n.d.	n.d.	n.d.	452.21	n.d.	n.d.	430.50
3400.31	n.d.	n.d.	n.d.	n.d.	n.d.	n.d.	929.16	n.d.	n.d.	**1778.67**
n.d.	n.d.	n.d.	n.d.	n.d.	n.d.	n.d.	406.77	n.d.	n.d.	**1031.63**
750.80	n.d.	n.d.	n.d.	n.d.	n.d.	n.d.	576.18	n.d.	n.d.	**537.27**
n.d.	n.d.	n.d.	n.d.	n.d.	n.d.	n.d.	571.56	n.d.	n.d.	**830.93**
3059.79	n.d.	n.d.	n.d.	n.d.	n.d.	n.d.	901.93	n.d.	n.d.	**604.57**
n.d.	n.d.	n.d.	n.d.	n.d.	n.d.	n.d.	364.92	n.d.	n.d.	**576.19**
2162.70	n.d.	n.d.	n.d.	n.d.	n.d.	n.d.	5787.09	n.d.	n.d.	**978.69**
84.74	n.d.	n.d.	n.d.	n.d.	n.d.	n.d.	3883.24	n.d.	n.d.	**833.31**
3053.07	n.d.	n.d.	n.d.	n.d.	n.d.	n.d.	n.d.	n.d.	n.d.	**1744.75**
37.28	n.d.	n.d.	n.d.	n.d.	n.d.	n.d.	192.13	n.d.	n.d.	311.32

Abbreviations: **MLS =** macrolide-lincosamide-streptogramin; **A-ERY =** anyhdro erythromycin; **AZI =** azithromycin; **CLA =** clarithromycin; **ERY =** erythromycin; **ROX =** roxithromycin; **SPI I =** spiramycin I; **SPI II =** spiramycin II; **SPI III =** spiramycin III; **CIP =** ciprofloxacin; **ENRO =** enrofloxacin; **NOR =** norfloxacin; **OXO =** oxolinic acid; **SARA =** sarafloxacin; **TIL =** tilmicosin; **ATC =** anhydrochlorotetracycline; **CTC =** chlorotetracycline; **OTC =** oxytetracycline; **TET =** tetracycline; **TYL =** tylosin; **A-SMX =** acetylsulfamethoxazole; **SCP =** sulfachloropyrazidine; **SPD =** sulfadiazine; **SDM =** sulfamethoxine; **SMR =** sulfamerazine; **SMZ =** sulframethazine; **SMI =** sulfamethizole; **SMX =** sulfamethoxazole; **SMT =** sulfamethoxydiazine; **STZ =** sulfathiazole; **TMP =** trimethroprim

Supplementary Table 9. Primers and quantitative polymerase chain reaction (qPCR) standard curve R^2 and efficiency values for each gene assay (average ± standard deviation).

Assay	Primer sequences (5'-3')	R^2	Efficiency[a]	References
16S rRNA	F: CGGTGAATACGTTCYCGG<br>R: GGWTACCTTGTTACGACTT	0.991±0.008	94.467±3.761	Suzuki, Taylor, & Long, 2000
*bla*TEM	F: TTCCTGTTTTTGCTCACCCAG<br>R: CTCAAGGATCTTACCGCTGTTG	0.981±0.011	100.667±11.150	Bibbal et al., 2000
*erm*B	F: GATACCGTTTACGAAATTGG<br>R: GAATCGAGACTTGAGTGTGC	0.995±0.004	87.825±4.155	Chen et al., 2007
*int*I1	F: CTGGATTTCGATCACGGCACG<br>R: ACATGCGTGTAAATCATCGTCG	0.995±0.002	84.425±7.988	Hardwick et al., 2008
sul1	F: CGCACCGGAAACATCGCTGCAC<br>R: TGAAGTTCCGCCGCAAGGCTCG	0.996±0.002	90.200±4.694	Pei et al., 2006
*van*A	F: GGGAAAACGACAATTGC<br>R: GTACAATGCGGCCGTTA	0.988±0.001	89.447±4.601	Dutka-Malen, Evers, & Coruvalin, 1995

[a]Efficiency = $10^{(-1/slope)}$-1.

References

Bibbal, D., Dupouy, V., Ferre, J. P., Toutain, P. L., Fayet, O., Prere, M. F., & Bousquet-Mélou, A. Impact of three ampicillin dosage regimens on selection of ampicillin resistance in *Enterobacteriaceae* and excretion of *bla*(TEM) genes in swine feces. *Appl. Environ. Microbiol.* 73, 4785–4790, doi:10.1128/AEM.00252-07 (2007).

Chen, J., Zhongtang, Y., Michel Jr., F. C., Wittum, T., & Morrison, M. Development and application of real-time PCR assays for quantification of *erm* genes conferring resistance to macrolides-lincosamides-streptogramin b in livestock manure and manure management systems. *Appl. Environ. Microbiol.* 73, 4405-4416, doi:10.1128/AEM.02799-06 (2007).

Dutka-Malen, S., Evers, S., & Courvalin, P. Detection of glycopeptide resistance genotypes and identification to the species level of clinically relevant enterococci by PCR. *J. Clin. Microbiol.* 33, 24–27, doi:10.1128/JCM.33.1.24-27.1995 (1995).

Hardwick, S. A., Stokes, H. W., Findlay, S., Taylor, M., & Gillings, M. R. Quantification of class 1 integron abundance in natural environments using real-time quantitative PCR. *FEMS Microbiol. Lett.* 278, 207–212 (2008).

Pei, R. T., Kim, S. C., Carlson, K. H., & Pruden, A. Effect of river landscape on the sediment concentrations of antibiotics and corresponding antibiotic resistance genes (ARG). *Water Res.* 40, 2427–2435 (2006).

Suzuki, M. T., Taylor, L. T., & DeLong, E. F. Quantitative analysis of small-subunit rRNA genes in mixed microbial populations via 5′- nuclease assays. *Appl. Environ. Microbiol.* 66, 4605–4614, doi:10.1128/AEM.66.11.4605-4614.2000 (2000).

Supplementary Table 10. Number of mapped reads per antibiotic resistance class in the influent and secondary effluent.

Sample	aminocoumarin	aminoglycoside	beta-lactam	elfamycin	fosfomycin	glycopeptide	MLS	multidrug
17-JUL-IN	191	2213	3286	0	61	148	9518	17311
17-JUL-SE	253	183	176	0	0	80	305	2751
17-OCT-IN	228	3249	4594	0	80	178	14178	24557
17-OCT-SE	103	925	379	0	2	50	744	3661
17-DEC-IN	230	2898	4653	0	72	150	12786	22628
17-DEC-SE	80	274	272	0	7	65	708	3820
18-FEB-IN	192	1743	2889	0	44	159	9082	17656
18-FEB-SE	77	474	413	0	14	28	865	5090
18-APR-IN	153	1375	2493	0	28	157	10724	14146
18-APR-SE	128	306	257	0	27	19	648	3823
18-JUN-IN	210	3543	4870	0	100	162	14991	27637
18-JUN-SE	94	529	843	1	6	64	1206	4720
18-AUG-IN	118	1719	2433	0	41	92	7666	12546
18-AUG-SE	241	457	347	0	5	44	800	3178
18-OCT-IN	8	242	364	0	8	11	1077	1883
18-OCT-SE	44	239	238	0	6	35	542	2278

Abbreviations: **MLS =** macrolide-lincosamide-streptogramin

other	peptide	phenicol	quinolone	rifamycin	sulfonamide	tetracycline	trimethoprim
427	3868	161	791	67	614	3873	181
236	293	31	49	143	183	251	17
613	5132	204	1364	102	879	5073	189
209	727	33	573	163	374	348	178
568	3696	223	1104	57	794	4913	198
204	474	27	89	161	202	417	21
658	2817	128	952	104	448	3877	143
218	495	29	178	164	135	199	21
370	2081	98	531	65	311	3838	72
271	358	18	98	128	122	265	12
542	4856	237	1282	107	953	6184	201
262	937	64	145	119	305	1007	58
293	2608	113	520	35	414	2851	116
194	553	26	156	130	210	439	99
36	328	20	78	4	53	450	22
121	284	11	88	90	202	231	28
